# Overcoming the Indirect Band Gap: Efficient Silicon Emission via Momentum-Engineered Photonic States

**DOI:** 10.1021/acs.nanolett.6c00596

**Published:** 2026-04-06

**Authors:** Aleksei I. Noskov, Alexander B. Kotlyar, Liat Katrivas, Zakhar Reveguk, Evan P. Garcia, V. Ara Apkarian, Christophe Galland, Eric O. Potma, Dmitry A. Fishman

**Affiliations:** † Department of Chemistry, 8788 University of California, Irvine, Irvine, California 92697, United States; ‡ George S. Wise Faculty of Life Sciences, 26745 Tel Aviv University, Tel Aviv 6997801, Israel; § Institute of Physics, 27218 Swiss Federal Institute of Technology (EPFL), CH-1015 Lausanne, Switzerland

**Keywords:** photon momentum, strong field confinement, silicon emission, photoluminescence

## Abstract

Silicon’s indirect band gap severely suppresses radiative recombination, limiting its use as an efficient light-emitting material. Although nanoscale confinement of carriers, dielectric resonators, or plasmonic structures can partially mitigate this limitation, these approaches typically require complex fabrication. Here we report a fundamentally different and scalable mechanism that enables efficient light emission directly from bulk silicon. By decorating a silicon wafer with ultrasmall (<2 nm) gold or copper particles, we observe intense luminescence spanning the visible and near-infrared. Remarkably, the emission is indistinguishable for Au and Cu decorations in both spectral and temporal domains, demonstrating that the confinement extent, not the material composition, governs the effect. We attribute the emission to spatially confined photonic states with broadened momentum distributions, enabling phonon-independent optical transitions otherwise forbidden in silicon. This mechanism yields quantum efficiencies comparable to direct-band-gap semiconductors and produces ∼10^5^-fold enhancement in the integrated emission intensity, establishing a practical route toward silicon light-emitting devices.

## Graphical abstract

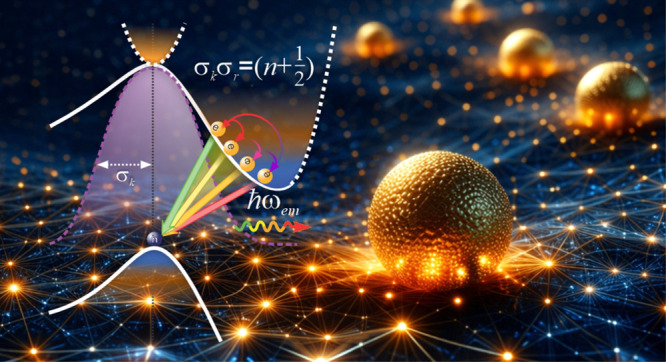

The cointegration of silicon electronics with photonic and optoelectronic components promises transformative advantages, including enhanced speed, increased bandwidth, and reduced power consumption. Silicon, buttressed by its natural abundance and well-established manufacturing infrastructure, is an important material for photonic integrated circuits.^1–3^ However, its indirect band gap compromises its optical properties. Unlike direct-band-gap semiconductors, optical transitions in silicon require phonon assistance to conserve momentum, as illustrated in Figure 1a. This reliance on phonons drastically reduces the rates of optical absorption and emission. Hot carriers rapidly thermalize to states near the band edge within 0.5 ps.^4–6^ Due to the slow nature of phonon-assisted radiation, a population bottleneck forms near the bottom of the conduction band, where electron–hole recombination is predominantly governed by nonradiative processes. Consequently, silicon exhibits a low luminescence quantum efficiency (η ∼ 10^–6^),^7^ severely limiting its use as a light emitter.

**Figure 1:**
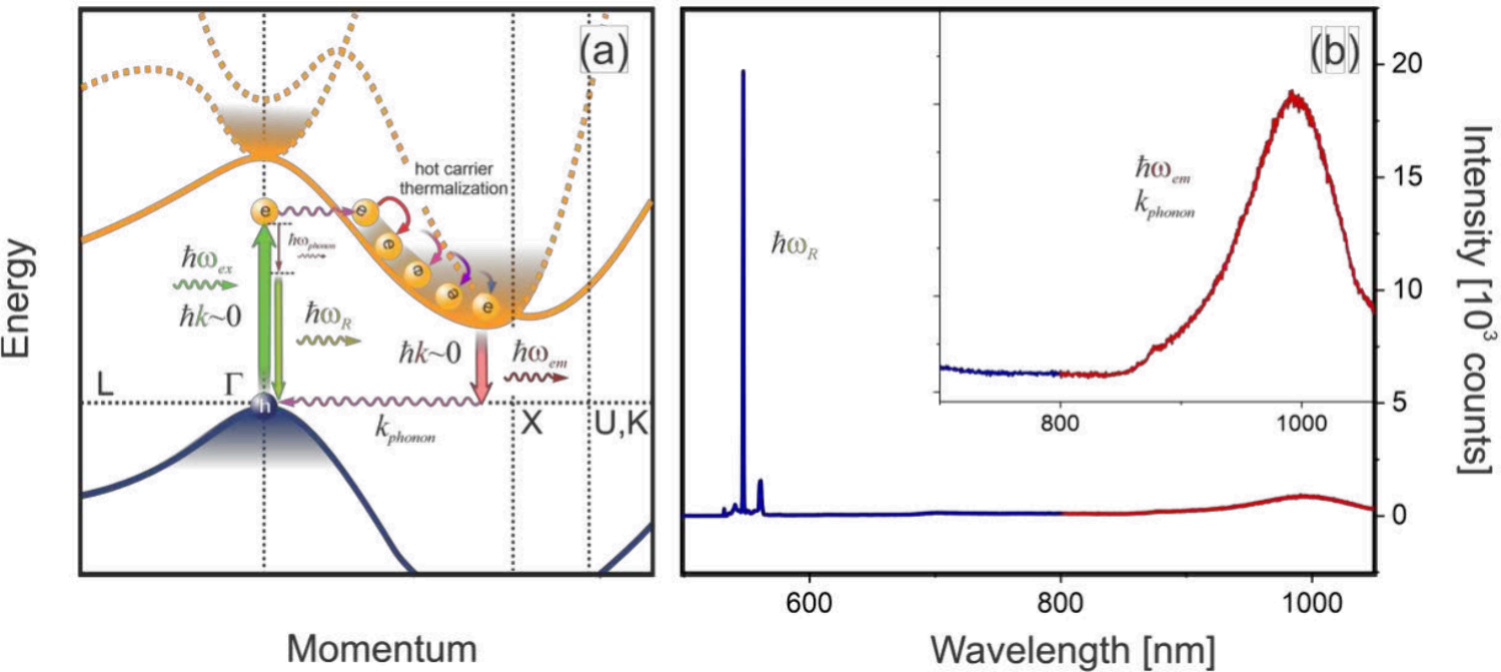
(a) Schematic of optical transitions in bulk silicon. Similar to absorption processes, transitions from the bottom of the conduction band require phonon assistance to conserve momentum, leading to the inherently low emission efficiency of bulk silicon. (b) Emission spectrum of bulk silicon, showing contributions from Raman scattering in the visible range (blue) and phonon-assisted emission from the bottom of the conduction band in the near-infrared spectral range (red). Excitation using 532 nm, 0.5 mW, and 0.75 NA.

Several strategies have been proposed to address silicon’s low quantum efficiency of light emission. For instance, reducing silicon’s dimensions to the nanoscale can increase its luminescence via quantum confinement.^8–12^ This effect is generally attributed to an increase in the overlap of electron and hole wave functions in momentum space, thereby accelerating radiative direct recombination across the indirect gap. Another approach utilizes (sub)­micrometer-sized silicon resonators, either as isolated structures or arranged in periodic arrays.^13–15^ These structures enhance light-matter interaction by leveraging optical resonances, which can increase radiative rates through the Purcell effect or sustain hot electron populations via the Auger effect. Plasmonic enhancements represent an additional avenue for improving silicon’s luminescent properties. Integrating silicon with plasmonic nanostructures takes advantage of their strong localized fields and high density of optical states.^16–19^ This approach has achieved notable improvements in silicon’s light emission efficiency, with reported quantum yields exceeding 1%.^16^

Despite recent advancements, practical on-chip silicon light sources remain elusive. This is largely due to fabrication complexities incompatible with existing circuit manufacturing processes. To overcome this, we require approaches that enhance silicon’s emission while minimizing material modifications. In this work, we address the fundamental limitation of silicon’s indirect-band-gap emission: the requirement for phonon-assisted transitions. We demonstrate that by eliminating the need for phonon involvement, silicon’s radiative rates can be dramatically enhanced without any alteration, structuring, or modification of the bulk material.

Figure 1b shows the emission spectrum of a clean silicon wafer following laser excitation at 532 nm. In addition to optical phonon Raman lines, the spectrum features weak emission near ∼1.0 μm, a phonon-assisted luminescence of rapidly thermalized electrons at the bottom of the conduction band. To overcome silicon’s inherently low radiative rates, we apply nanometer-sized gold and copper particles to the silicon’s surface. This simple and direct procedure remarkably changes the wafer’s emission spectrum in a particle size-dependent manner, as shown in Figure 2a. For 5 and 15 nm Au particles, the phonon-assisted luminescence near the silicon band edge remains visible, while an additional spectral band appears in the 600–700 nm range. When the Au nanoparticle size is reduced to 1.2 nm (Supporting Information Part I), the band-edge luminescence from silicon is fully suppressed and a bright, broadband emission emerges, spanning the entire spectral range from the 532 nm excitation wavelength to approximately 1.0 μm. Nearly identical emission profiles are observed when the silicon surface is decorated with 1.2 nm Cu nanoparticles, indicating that the effect is predominantly dictated by particle size and that the chemical differences between Au and Cu are less of a factor. The observed emission cannot be attributed to intrinsic luminescence from either gold (Figure 2, purple line, and the discussion in Supporting Information Part V) or copper. Instead, it points to the activation of new radiative channels in silicon.

**Figure 2:**
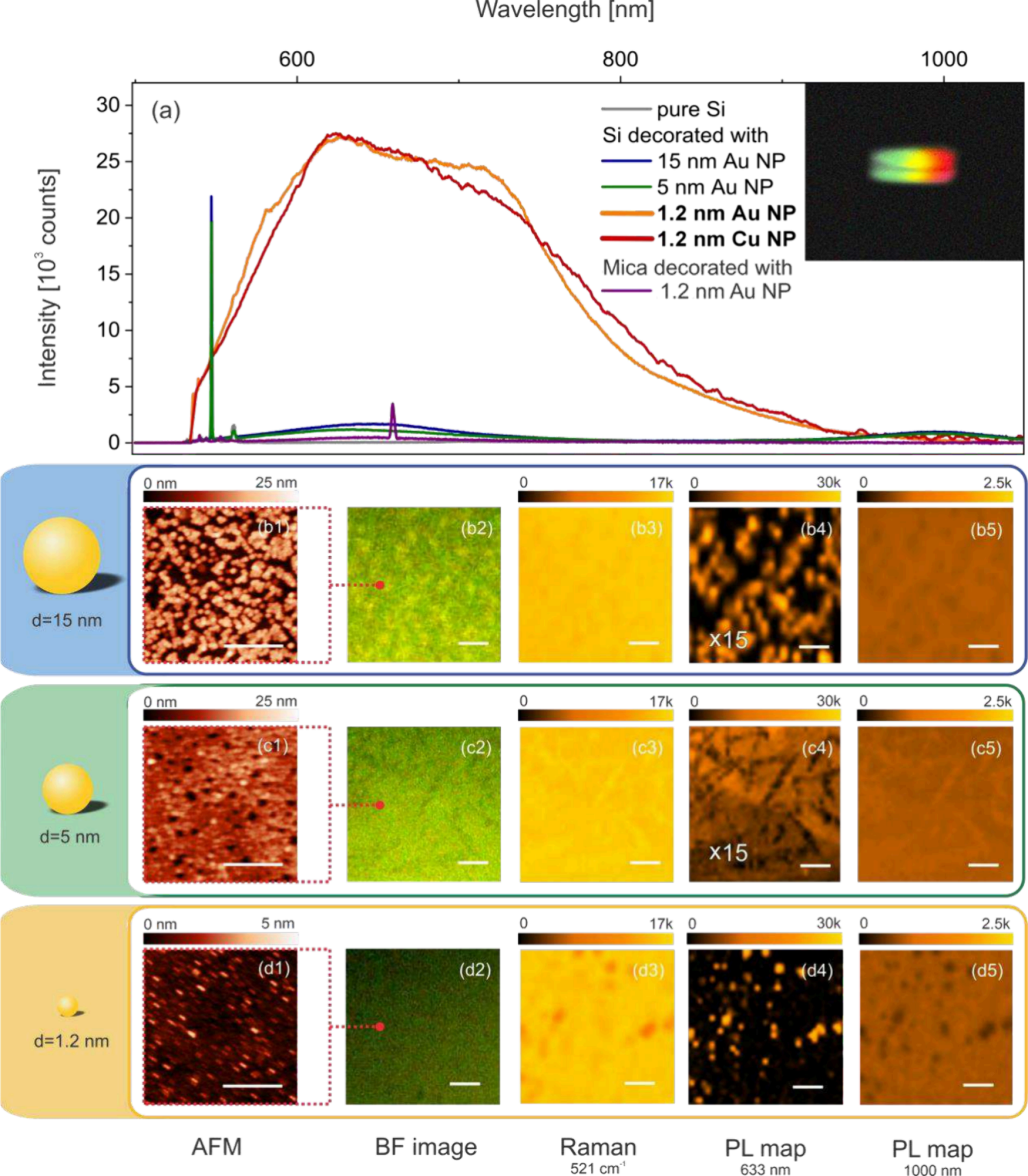
(a) Emission spectra of a pure silicon wafer (gray) and wafers decorated with gold and copper nanoparticles: 15 nm Au (blue), 5 nm Au (green), 1.2 nm Au (orange), and 1.2 nm Cu (red, normalized to orange by factor of 0.7). Excitation using 532 nm, 0.5 mW, and 0.75 NA. The emission spectrum of 1.2 nm Au particles on mica, representing the intrinsic emission of the nanoparticles themselves and Raman of mica substrate, is shown as the purple spectrum. The inset shows a side view of the emitting silicon wafer captured on a phone camera (10 s exposure) through an Amici prism (see also Figure 4c). Characterization and imaging of wafer surfaces decorated with 15 nm (b), 5 nm (c), and 1.2 nm (d) gold particles: (1) AFM maps (scale bar 400 nm); (2) bright-field optical images using white-light illumination; (3) Raman maps at the 521 cm^–1^ silicon phonon line; (4) PL maps at 630 nm; (5) PL maps at 1000 nm, representing silicon’s phonon-assisted luminescence from the bottom of the conduction band at X point. The scale bar is 5 μm for all optical images. Note: PL maps at 630 nm for 15 nm (b4) and 5 nm (c4) samples are enhanced by a factor of 15.

To further examine the nature of the sample’s emission characteristics, we performed spatially resolved measurements of the particle-decorated silicon wafers. The atomic force microscopy (AFM) maps in Figure 2b1–d1 confirm the presence of the gold particles on the wafer’s surface, forming a monolayer configuration. For the 15 nm particles, the bright-field reflection image in Figure 2b2 reveals faint structures, indicating weak, but visible, plasmon-enhanced scattering activity (see Figure SF3 in Supporting Information Part II). Scattering is noticeably weaker for the 5 nm particles, as shown in Figure 2c2, and becomes undetectable for the 1.2 nm Au clusters (Figure 2d2). For these smallest particles, plasmons are overdamped, and scattering is of the Rayleigh type. These experiments underline that, unlike their larger counterparts, the 1.2 nm particles, either Cu or Au (Figures SF4 and SF5), have no significant effect on the scattering properties of the Si surface and are therefore invisible to the naked eye.

The Raman maps, recorded at the 521 cm^–1^ optical phonon line of silicon, appear largely unaffected by the 5 and 15 nm particles deposited on the wafer’s surface, as shown in Figure 2b3,c3. For the 1.2 nm decorations, however, the intensity of the silicon’s Raman response is reduced, as shown for the patch of particles in Figure 2d3, and consistent with increased optical absorption in the Si surface layer (as discussed further below). A similar trend is seen in the PL maps at silicon’s band edge, which are immune to the presence of the larger particles (Figure 2b5,c5), but show locally depleted signals in specific regions, when 1.2 nm particles are used (Figure 2d5). On the other hand, the location of the Au particles can be clearly seen in the PL map at the 630 nm emission wavelength, with the strongest signals observed for the 1.2 nm particles (Figures 2b4–d4). Similar strong emission is observed for wafers decorated with 1.2 nm Cu particles (Figures 2a and SF8 and SF9 in Supporting Information Part III and discussion therein). Together, these maps show that sub-2-nm Au or Cu particles induce new emission across the visible and near-IR spectral range, while the corresponding surface decoration does not produce meaningful Raman enhancement and instead strongly suppresses the phonon-assisted photoluminescence (PL) at the silicon band edge. Notably, for both 1.2 nm Au and Cu particles, the PL maps appear spatially heterogeneous (“patchy”) across the surface. This spatial heterogeneity does not reflect large-scale variations in surface coverage, but rather the sensitivity of the effect to local particle density and proximity to the surface (see Supporting Information Part III for further discussion) at the nanometer scale. In particular, this behavior suggests that the interaction responsible for the emission is short-ranged and depends on the relative positioning of multiple nanoparticles proximal to the silicon surface, consistent with the confined optical fields reported in Ref. 20. As a result, only selected regions of the surface satisfy the conditions necessary for efficient emission, leading to the observed “patchy” PL patterns.

Experiments with 5 and 15 nm Au particles on mica exhibit emission features similar to the 600–700 nm band observed in Figure 2a, confirming gold PL^21–24^ as the source of this visible emission (see Supporting Information Part V for a discussion on the signal’s origins from pure Au structures). However, the gold PL from 1.2 nm Au particles on both mica and silicon is significantly different in terms of both spectral shape and strength of the signals (see Figures SF11–SF14 in Supporting Information Part IV), ruling out gold luminescence as the primary mechanism responsible for the strong broadband emission observed with 1.2 nm Au particles on silicon. This also agrees well with solution batch PL experiments, where no discernible emission is observed for high concentration bath solutions for both 1.2 nm Au and Cu nanoparticles (see Figures SF19–SF23 in Supporting Information Part VI). Furthermore, the intensity of visible luminescence from silicon wafers decorated with 1.2 nm Au particles remains unchanged regardless of the number of applied particle layers. Even with ∼40 layers, the luminescence intensity remains comparable to that of a single monolayer (Supporting Information Part VII). This observation strongly suggests that the luminescence originates solely from the Au–Si interface and does not scale with the overall gold content beyond the initial monolayer. The low scattering activity and overdamped plasmonic properties of the 1.2 nm Au particles further rule out surface plasmon enhancement effects as the origin of the observed bright, broadband luminescence. On the basis if these findings, we conclude that gold PL cannot account for the bright visible emission observed with 1.2 nm Au particles on silicon, indicating the presence of a distinct mechanism.

We note that the existence of an interfacial state between the metal nanoparticle and the semiconductor has been discussed extensively in the literature.^25^ Our measurements, however, do not conclusively point to a prominent role for such states in the PL process. First, metal–semiconductor interfacial states are also expected for the larger (5 and 15 nm) Au particles, yet their emission spectra are of a markedly different shape and brightness than in the case of 1.2 nm particles. This suggests that a fundamentally different mechanism is at play for the smallest particles. Second, the luminescence generated by interfacial states is expected to be sensitive to the metal’s chemical makeup. The observed similarity between the PL spectra of Au or Cu decorated silicon wafers thus renders the direct involvement of an interfacial state rather unlikely.

The unique optical properties of the Si-particles interfacial region are further demonstrated in Figure 3. Figure 3a compares the Raman optical phonon signature from pure silicon and silicon with 1.2 and 15 nm Au and 1.2 nm Cu particles. When the 15 nm Au particles form larger clusters, creating accidental plasmonic hotspots, an enhancement of the Raman signal is observed. However, when the particles are assembled into intact monolayers, the Raman signal remains unchanged compared to bare silicon. In contrast, the presence of 1.2 nm Au or Cu particles strongly suppresses the inherent Raman signal from silicon. The suppression of the Raman signal suggests enhanced light absorption at the interface between bulk Si and the particle, leading to reduced illumination of the underlying silicon volume.

**Figure 3:**
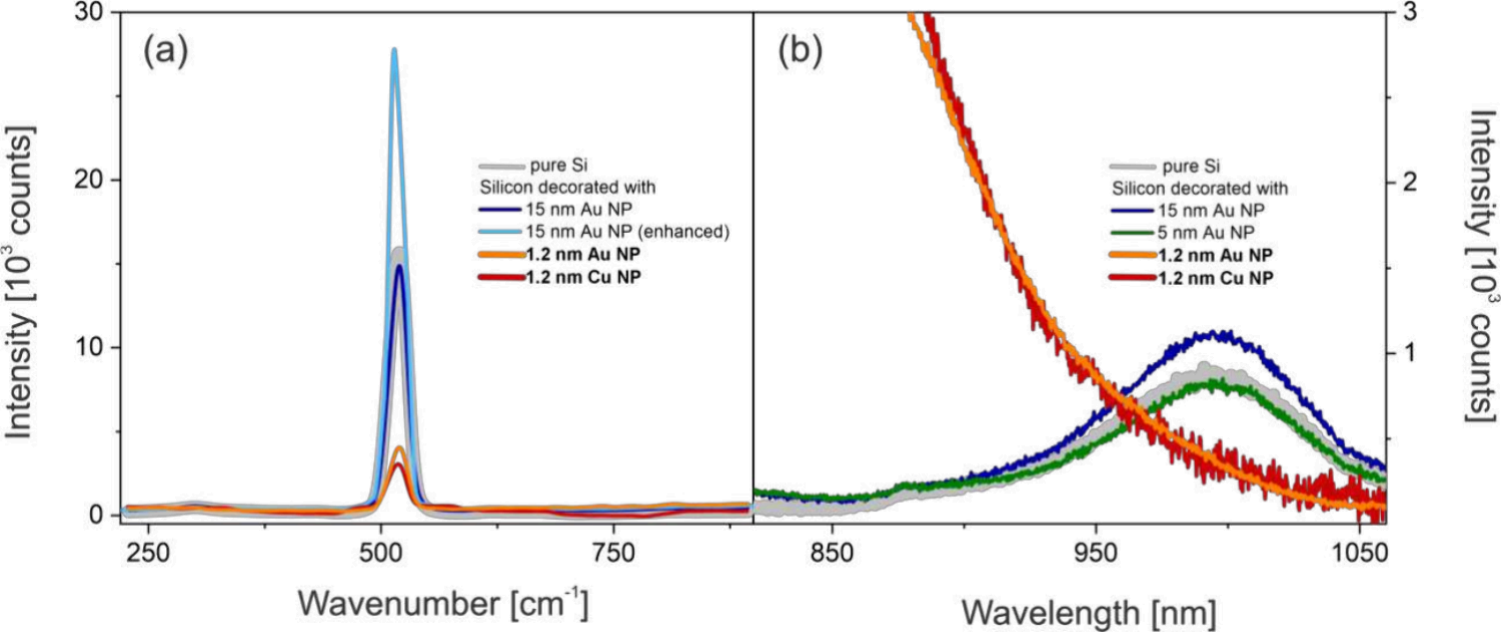
(a) 521 cm^–1^ Raman line of crystalline silicon. Larger particles show a modest surface enhancement (light blue line), while areas with intact 1.2 nm particles show strong suppression of silicon’s Raman signal. Excitation using 532 nm, 0.5 mW, and 0.75 NA. (b) PL in pure Si originates from phonon-assisted transitions from the bottom of the conduction band. The PL is fully suppressed when 1.2 nm particles are present.

Enhanced light absorption in silicon decorated with nanometer-sized particles has previously been reported and attributed to highly confined optical states.^20,26,27^ According to Heisenberg’s uncertainty principle, strong spatial confinement of light leads to a broadened distribution of photonic momenta, which, for sufficiently small particles, can span the entire Brillouin zone (see also Supporting Information Part VII).^28–30^ These momentum-broadened optical states enable photon-induced indirect transitions in silicon without phonon assistance, significantly accelerating the absorption process. More importantly, multiple independent measurements of the enhanced absorption rates consistently indicate that the origin of the observed effects lies in silicon itself.^20^ First, when sub-2-nm photon confiners are present atop a silicon photodiode, a significant increase in the photogenerated current is observed, thus indicating enhanced electron–hole pair generation *within* the silicon material. Second, localized heating of silicon microstructures to temperatures exceeding 1500 °C when placed in proximity to a nanometric photon confiner points to enhanced light absorption in silicon, as this condition cannot be achieved through absorption in the metal nanoparticle alone.

From the observed suppression of the Raman signal, we find that the effective absorption coefficient near the Si surface has increased *up to 3 orders of magnitude*, consistent with experiments reported in a previous study.^20^ We note that, in contrast to the localized high-temperature conditions reported in ref 20 for tip-based geometries, the present planar bulk configuration does not exhibit measurable Raman peak shifts or broadening (Figure 3a), indicating that the silicon remains near room temperature under our experimental conditions (see Supporting Information Part III and Figure SF10). This relaxed momentum conservation requirement is also evident in surface-enhanced Raman scattering (SERS) experiments on gold plasmonic antennas, where it contributes to the observed broad electronic Raman background of the metal.^31–35^ We propose that these momentum-broadened optical states play a crucial role in enhancing radiative rates for PL in silicon as well, leading to the observed bright emission across the visible spectrum.

Figure 3b shows that phonon-assisted radiative recombination near the bottom of the conduction band remains intact or even enhanced for 5 nm and 15 nm Au particles. However, when either Au or Cu single-layer 1.2 nm particles are deposited onto the wafer surface, this recombination pathway is almost completely suppressed. This observation suggests that a new, highly efficient radiative channel emerges, one that outcompetes both intrinsic radiative and nonradiative relaxation processes near the band edge. We note that the red tail emission for both the Au and Cu decorated silicon disappears exactly at silicon’s band edge energy, as shown enlarged in Figure 3b. This observation further suggests that the luminescence originates from silicon and not from the individual particles.

We attribute this new radiative channel in silicon to momentum-broadened optical states that enable direct recombination of conduction electrons with holes in the valence band along a broad range of momentum pathways. Figure 4a illustrates a representative example of the transition scenario involving holes near the Γ point only. Upon visible light excitation, electrons undergo ultrafast relaxation (<0.5 ps) to the bottom of the conduction band through electron–electron and electron–phonon scattering processes.^4–6^ Full thermalization with the lattice is completed over longer time scales, typically a few picoseconds and up to 1 ns at high density due to the formation of a hot-phonon bottleneck. Once thermalized, the photoexcited electron population in bulk silicon must remain trapped, depleting slowly via phonon-assisted radiative and intrinsic nonradiative relaxation processes on time scales of tens of microseconds.

**Figure 4:**
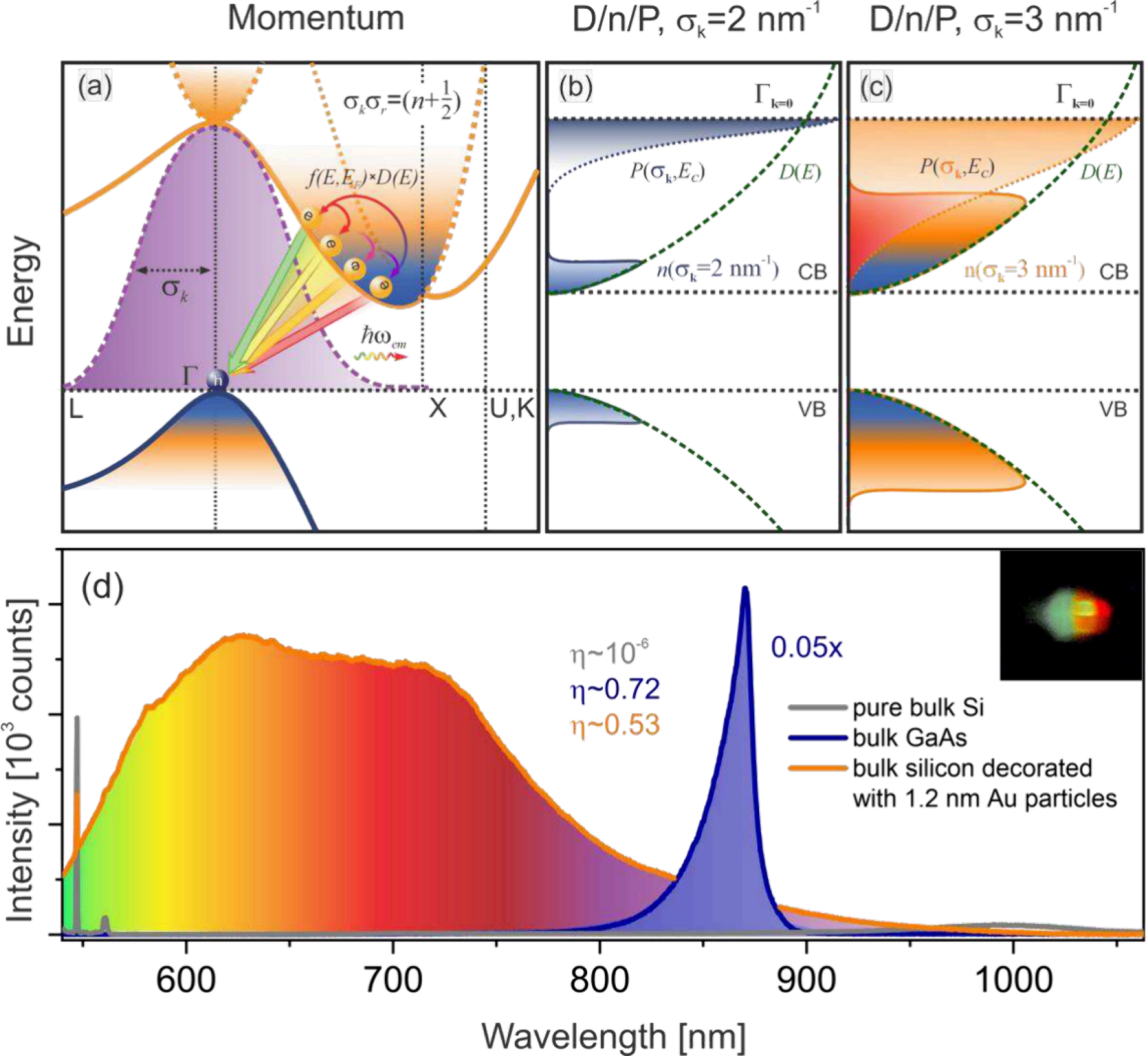
(a) Schematic illustration of optical transitions in bulk silicon decorated with sub-2-nm Au or Cu nanoparticles. Momentum-enhanced optical absorption drives a high-density electron gas into the conduction band. As the band fills, electron population redistributes toward higher-energy states. (b) Calculated electron distribution and corresponding transition probability function for σ_k_ = 2 nm^–1^. (c) Both functions broaden significantly with decreasing confinement size (i.e., increasing σ_k_). For σ_k_ > 2 nm^–1^, the resulting spectral overlap provides an efficient pathway for ultrafast radiative depletion of the conduction band population, giving rise to broadband emission. (d) Emission spectra of bare silicon, silicon decorated with 1.2 nm Au nanoparticles, and bulk GaAs measured under identical conditions. Comparison with GaAs luminescence indicates an external quantum efficiency exceeding 0.5 for the decorated silicon. Inset: Strong broadband emission from the decorated silicon wafer, visible to the naked eye. The emission is viewed through an Amici prism and recorded using an iPhone camera under 532 nm excitation with 10 s integration time (see Figure SF33 in Supporting Information Part XII).

This paradigm shifts entirely when confined photonic states are introduced. This effect can be understood in terms of the local photonic density of states at the particle–silicon interface, where strong spatial confinement gives rise to optical modes with a broadened momentum distribution. In this view, the interface defines a continuum of vacuum modes with an extended wavevector spectrum, providing momentum components that enable electronic transitions that would otherwise be momentum-forbidden, i.e., indirect transitions in bulk silicon. For a given spatial confinement of variance σ_r_, the photon acquires an expanded momentum spectrum of variance σ_k_ (Figure 4a, purple Gaussian profile).^20^ This relationship follows directly from the quantum uncertainty principle $\sigma_{r}\sigma_{k}=n+1/2$ , where *n* is the expectation value of the occupation number operator *N̂* = *a* ^†^ *a* (see Supporting Information Part VIII for additional discussion). The expansion of photon momentum under sub-2-nm confinement leads to a dramatic enhancement in silicon’s optical absorption, with estimated increases of up to 3 orders of magnitude, as we previously demonstrated experimentally.^20^ In our current experiments, this effect is evidenced by the pronounced depletion of the intrinsic Si–Si Raman line observed beneath both Au and Cu nanoparticles with diameters of *d* = 1.2 nm (σ_r_ ∼ *d*/2.355 = 0.5 nm; σ_k_ = 3 nm^–1^). This observation has significant implications. Although the incident photon fluxes are modest, typically yielding an estimated conduction-band electron density of ∼10^19^ cm^–3^ for freely propagating light, the enhanced absorption, driven by momentum-expanded photonic states (σ_k_), is expected to proportionally increase the conduction band carrier density *n*_CB_. A detailed discussion and modeling of how momentum-enabled absorption enhancement impacts the conduction-band electron density are provided in Supporting Information Part IX.

At sufficiently high electron densities in the conduction band (>10^20^ cm^–3^), the system enters a highly degenerate electron–hole plasma regime,^36–38^ characterized by a Fermi energy that rises well above the conduction band edge. In this regime, electrons begin to significantly fill the conduction band valleys, forcing the population to spread into higher-energy states. Representative electron distributions for σ_k_ = 2 and 3 nm^–1^ are shown in Figure 4b,c (see also Figure SF26).

Simultaneously, momentum-expanded photonic states enable a new radiative channel, with the transition probability *P*(σ_k_) defined as the projection of the Gaussian photon momentum distribution onto the conduction band dispersion profile. This probability is highest for electrons occupying elevated conduction band states (Figure 4b) and broadens significantly toward the band edge for photon confinement below 2 nm (σ_k_ > 1.7 nm^–1^; Figures 4c and SF27). The broadening of both the electron distribution and the radiative transition probability as a function of σ_k_ results in a substantial spectral overlap between the two. This overlap establishes a viable pathway for radiative depletion of the conduction band and facilitates the emission of broadband radiation. Notably, this strong dependence of radiative recombination on local carrier density also provides a natural explanation for the spatially heterogeneous (“patchy”) emission patterns observed in Figure 2d4 (see also Figures SF6b3 and SF7c4 in Supporting Information Part III), as only regions reaching sufficiently high excitation densities contribute efficiently to the emission. A natural consequence of this model is that electrons occupy increasingly higher states in the conduction band when the incident optical intensity is increased, predicting an effective blueshift of the emission maximum. This is indeed observed, as the center of mass of the emission spectrum shifts markedly toward higher energies with increasing input flux (Figure SF29). While the red side of the emission spectrum remains nearly unchanged with varying excitation power, the observed blue shift of 0.2–0.3 eV is consistent with expectations for electron densities exceeding 10^21^ cm^–3^ (see Figure SF29 in Supporting Information Part IX and discussions therein), further supporting the confinement-dependent mechanism. As expected, when the small particles within the focal spot melt or coalesce into larger clusters under intense and prolonged illumination, they can no longer sustain the required momentum expansion for these transitions. As a result, the system reverts to displaying the original Raman and phonon-assisted PL features characteristic of bulk silicon (Figures SF30 and SF31 in Supporting Information Part X).

Since the newly opened radiative channel should effectively render the transition direct (phononless), substantially increasing the transition probability, the radiative rates are expected to be accelerated by more than 3 orders of magnitude relative to conventional silicon. Consequently, the PL lifetime is significantly reduced, from the typical microsecond range to the nanosecond regime. Figure SF15 presents the time-resolved PL data for 1.2 nm Au and 1.2 nm Cu particles on a clean silicon wafer. Both cases yield lifetimes of approximately ∼2 ns. In the context of our model, this indicates that the decay dynamics are fully dominated by the newly opened radiative channel. When either 1.2 nm Cu or 1.2 nm Au clusters are placed in close proximity to the silicon surface, the resulting emission exhibits indistinguishable shapes in both the frequency domain (Figures 2 and SF11–SF14) and the time domain (Figure SF15 in Supporting Information Part IV). This identical behavior provides additional strong evidence that the emission mechanism is relatively insensitive to the material properties of the confiner and must therefore originate from silicon itself.

Our results confirm that the newly enabled radiative transition pathways efficiently deplete the conduction band electron population in bulk silicon, effectively bypassing conventional radiative and nonradiative loss mechanisms and yielding high quantum efficiency emission. To quantify this efficiency, we calibrated the enhanced silicon emission intensity against the 865 nm PL peak of undoped bulk GaAs, used here as a reference standard (Figure 4d). On the basis of this analysis, we estimate an external quantum efficiency of approximately η ≈ 0.53, comparable to that of direct-band-gap GaAs (η_GaAs_ ∼ 0.72)^39,40^ (see Table 1 in Supporting Information Part XI for details). Despite being confined to the Si interface, this high quantum efficiency results in a sufficiently bright emission that is readily visible to the naked eye, as shown in Figure 4d (see also Figure SF33).

These findings demonstrate that by carefully engineering confined optical states, silicon—traditionally a material with inherently low emission efficiency—can be transformed into a highly efficient light emitter. In this study, sub-2-nm Au or Cu nanoparticles were used to induce extreme photon confinement at the silicon surface, revealing that particle size, rather than chemical identity, is the key parameter governing this transformation. Importantly, the enhancement of light emission occurs without altering the bulk silicon wafer, its crystal or electronic structure, indicating that the observed emission enhancement arises primarily from the altered photonic environment.

The concept of momentum-broadened optical states challenges the long-standing assumption that silicon’s optical behavior is fundamentally limited by its indirect band gap. The results presented here open new avenues for the design and realization of advanced silicon-based optical and optoelectronic components. Moreover, the photonic phenomenon holds potential to enable otherwise forbidden optical transitions across a broad family of momentum-forbidden materials, such as bulk GaP and MoS_2_, as well as other two-dimensional van der Waals transition metals and main-group chalcogenides, and even one-dimensional van der Waals pnictogen chalcogenides.^41^

## Supplementary Material



## Data Availability

The data are available from the corresponding author upon reasonable request.

## Notes

Additional discussions on the synthesis of nanoparticles, linear spectroscopy of nanoparticle coatings, electron microscopy characterizations, additional data and discussion of microscopic characterizations of the decorated wafers, Raman thermometry, spectral and temporal characterization and comparison for different particle sizes, additional discussions on the origin of the emission from ordered and individual, but highly packed gold nanoparticles, spectroscopic analysis of the chemical ligand involved in synthesis, additional discussion on the confined field origin and Heisenberg uncertainty principle, additional power dependence experiments and modeling of distribution of highly degenerate electron gas and momentum-expansion, additional data on particle damage under high and/or prolonged flux, and discussion on the quantum yield estimation (PDF)
