# Supplementary material for: Overcoming the Indirect Band Gap: Efficient Silicon Emission via Momentum-Engineered Photonic States
Source: Nano Lett. 2026 Apr 6;26(15):5187–94. doi: 10.1021/acs.nanolett.6c00596 (PMC13107516; doi:10.1021/acs.nanolett.6c00596)
Supplement: Supplementary file 1 [file nl6c00596_si_001.pdf]

Overcoming the indirect bandgap: efficient silicon emission via momentum-engineered photonic states

Aleksei I. Noskov[1], Alexander B. Kotlyar[2], Liat Katrivas[2], Zakhar Reveguk[2], Evan P. Garcia[1], V. Ara Apkarian[1], Christophe Galland[3], Eric O. Potma[1], Dmitry A. Fishman[1]*

[1]*Department of Chemistry, University of California, Irvine, Irvine, CA 92697, USA*

[2]*George S. Wise Faculty of Life Sciences, Tel Aviv University, Tel Aviv 6997801, Israel*

[3]*Institute of Physics, Swiss Federal Institute of Technology (EPFL), CH-1015 Lausanne, Switzerland*

Part I. Nanoparticle synthesis and deposition.

Synthesis of 1.2 nm nanoparticles (NAD-NP). 300 µl of 100 mM aqueous nicotinamide adenine dinucleotide (NAD) solution was incubated with 80 mM $AuHCl_4$ at ambient temperature for 15 min. The mixture was added to 30 mL of freshly prepared aqueous solution containing 8 mM KOH and 1.5 mM $NaBH_4$ under constant high-speed stirring. An intensive dark brown color appeared, indicating nanoparticle formation. The solution was stirred for an additional 5-10 min. The particles were then centrifuged for 5 min at 20°C in two 15 mL 50 kDa Amicon-Ultra Centrifugal Filter Units at 4000 rpm. The filtrate was transferred into two 15 mL 10 kDa Amicon-Ultra Centrifugal Filter Units and centrifuged at 4000 rpm for 15 min. The retentate fractions (that did not pass through an ultrafiltration unit), containing concentrated particles (0.2–0.3 mL from each filtration unit), were pooled together. The pooled retentate was diluted into 15 mL of double-distilled water (DDW) and centrifuged in a 15 mL 10 kDa Amicon Ultra Centrifugal Filter Unit as described above. The centrifugation/dilution cycle was repeated four times to ensure complete removal of unbound NAD. After the last centrifugation/dilution cycle, the final retentate fraction (~0.2-0.3 mL) was collected. The absorbance of the particles at 420 nm was commonly equal to ~100 AU. The particles are stable and can be stored either at 4 ºC of 25 °C for at least one month.

Figure SF1 shows an HR-TEM image that was obtained with a Thermo Fisher Scientific Talos F200i transmission electron microscope instrument. The sample was prepared by dropping 4 µL of NAD-NPs solution (absorbance at 420 nm = 3 AU) on an ultrathin (3–4 nm) carbon-coated copper grid. After 1 min, the solution was pulled out by touching the edge of the grid with filter paper.

Synthesis of 5 nm and 15 nm Au nanoparticles. 5 nm and 15 nm Au nanoparticles (Au-NP's) were synthesized essentially as described in [1,2]. 30 mL of the 15-nm particles was centrifuged for 5 min at 20 °C in a 15 mL 100 kDa Amicon-Ultra Centrifugal Filter Units at 2000 rpm. The final retentate fraction (~0.2 mL) was collected. 30 mL of 5-nm Au-NP's was centrifuged for 15 min at 20 °C in a 15 mL 10 kDa Amicon-Ultra Centrifugal Filter Units at 4000 rpm. The final retentate fraction (~0.2 mL) was collected. The absorbance of both types of particles at 520 nm was ~ 70 AU. The particles can be stored under ambient conditions for months.

Deposition of 1.2 nm NAD-NP's on silicon. A crystalline silicon wafer (<100>, undoped, 280 µm thickness) was treated with 5% HF for 5 min. The acid was thoroughly removed by rinsing with DDW, and the surface was dried by a flow of nitrogen gas. A 10-20 µL drop of NAD-NPs (OD ~ 100 AU at 420 nm) in 0.3M KCL was applied to the surface and left on it for 15 hours in a humid atmosphere. The surface was then rinsed with cold DDW and dried with a nitrogen gas flow.

Deposition of 1.2 nm nanoparticles on mica. A 10-20 µL drop of NAD-NP's (OD ~ 100 AU at 420 nm) in 0.2M KCL was applied to a freshly cleaved mica and left on it for 15 hours in a humid atmosphere. The surface was then rinsed with cold DDW and dried with a nitrogen gas flow.

Deposition of 15 nm and 5 nm Au nanoparticles on silicon. A crystalline silicon wafer (<100>, undoped, 280 µm thickness) was treated with 5% HF for 5 min. The acid was thoroughly removed by rinsing with DDW, and the surface was dried with a flow of nitrogen gas. A 10-20 µL drop of 15-nm Au-NP's (OD ~ 50 AU at 520 nm) in 50 mM KCL and 5 mM Bis(p-sulfonatophenyl)phenylphosphine dihydrate dipotassium salt (BSPP) or 5-nm Au-NP's (OD ~ 50 AU at 520 nm) in 150 mM KCL and 5 mM BSPP was applied to the surface and left on it for 15 hours in a humid atmosphere. The surface was then rinsed with cold DDW and dried with a nitrogen gas flow.

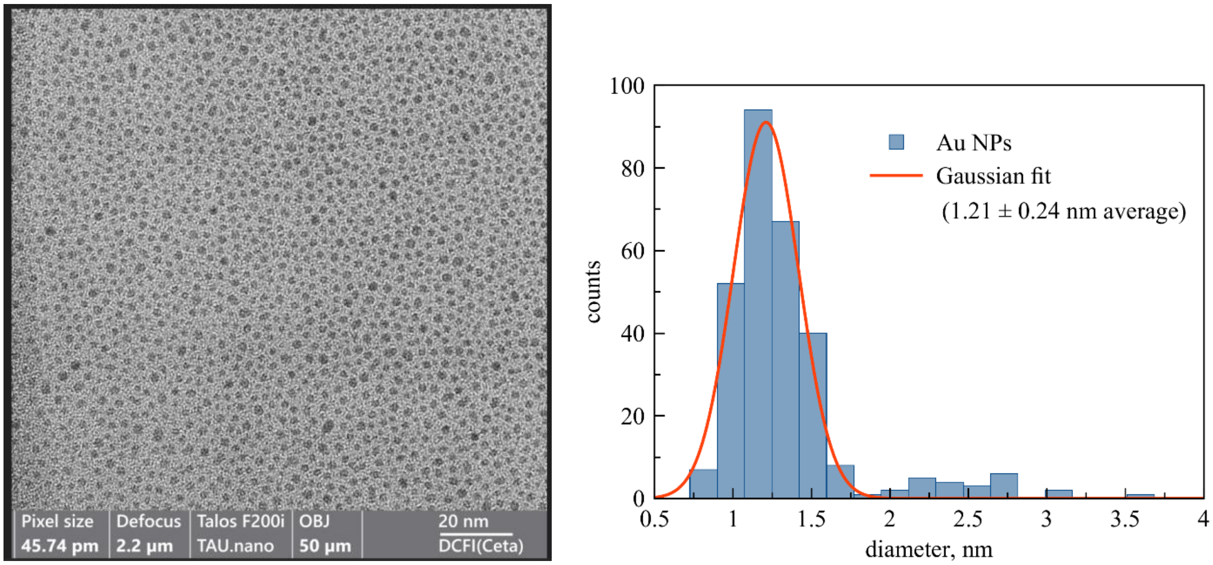

Figure SF1. HR-TEM characterization of 1.2 nm Au nanoparticles.

Preparation of Ultrasmall Copper Nanoparticles (CuNPs). ATP (0.125 M) was mixed with $CuSO_4$ (0.125 M) in 0.3 mL of DDW and incubated at room temperature for 10 minutes. The resulting mixture was added to 30 mL of 2.5 mM $NaBH_4$ solution in DDW under vigorous stirring. The solution rapidly turned brown, indicating the formation of CuNPs. Stirring was continued for an additional 5 minutes.

The reaction mixture was then centrifuged at 20 °C for 5 minutes using 15-mL 50 kDa Amicon Ultra centrifugal filter units at 4000 rpm to remove larger particles, which were retained by the filter and discarded. The filtrate, containing smaller nanoparticles, was further concentrated by centrifugation at 20 °C for 15 minutes using 10 kDa Amicon Ultra centrifugal filters. The retained fraction (~0.3 mL) was subsequently chromatographed on a gel filtration NAP-10 column equilibrated with 10 mM HEPES-K buffer (pH 7.5). The void volume fraction (~0.5 mL), containing CuNPs, was collected. To stabilize the nanoparticles, 5 µL of 0.1 M ATP was added to the eluate. The final preparation typically has a volume of ~0.5 mL and OD of ~50 at 420 nm. The CuNP solution was saturated with argon gas and frozen at –80 °C. Frozen samples can be stored for several months without noticeable changes in their absorption spectrum or physicochemical properties.

AFM characterization of CuNPs. CuNPs were diluted in 0.1 M KCl to a final absorbance of approximately 20 mAU at 420 nm. A 20-µL aliquot of the diluted sample was deposited onto a freshly cleaved mica substrate and incubated under ambient conditions for 30 seconds. The surface was then rinsed with 1 mL of ice-cold DDW and rapidly dried under a stream of nitrogen gas. AFM imaging was conducted using a Solver PRO system (NTEGRA SPECTRA II, NT-MDT Ltd., Moscow, Russia) operated in semi-contact (tapping) mode. High Accuracy Non-Contact AFM probes from the PHA-NC series (ScanSens, Munich, Germany), with a resonance frequency range of 70–180 kHz, were used. Acquired images were flattened using Nova image processing software (NT-MDT Ltd., Moscow, Russia) by fitting each scan line to a second-order polynomial and subtracting the fitted line from the original data. Quantitative analysis of the images was performed using Gwyddion software (http://gwyddion.net/).

HR-TEM characterization of CuNPs. HR-TEM images were acquired using a Thermo Fisher Scientific Talos F200i transmission electron microscope (ThermoFisher Scientific, USA) in 200 kV bright filed mode. Samples were prepared by depositing 1.5 µL of the CuNP solution

(absorbance at 420 nm ~25) onto ultrathin (3–4 nm thickness) carbon-coated copper grids, which had been pretreated with a 25% O_2 / 75% Ar plasma. After a 2-minute incubation, excess solution was removed by gently touching the edge of the grid with filter paper. The grids were then dried under low vacuum for 15 minutes. Following drying, the samples were briefly (10 seconds) exposed to an O_2/Ar plasma to remove residual organic material.

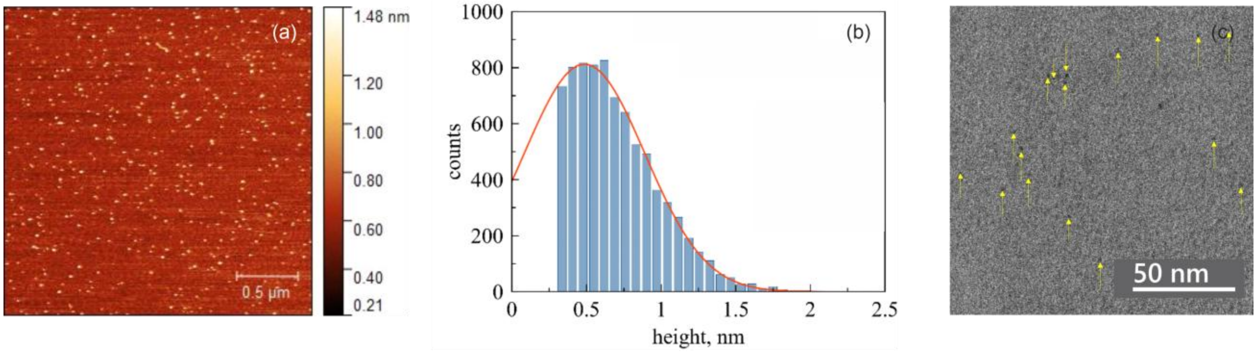

Figure SF2. (a) AFM topography of Cu nanoparticles on a mica surface. The particles were deposited on a freshly cleaved mica and scanned as described in Materials and Methods. The inset presents a height analysis of the particles by Gwyddion software (http://gwyddion.net/). (b) Corresponding height distribution of Cu nanoparticles derived from AFM measurements. (c) HR-TEM characterization, showing a size distribution in the range of 1-1.5 nm. Due to rapid disintegration of the particles under high-energy electron beam exposure, accurate size determination is challenging. The particles appear as small dark spots and are marked with yellow arrows for easier identification.

While AFM characterization reveals a Poisson-like size distribution with a width extending slightly above 1 nm, the HR-TEM measurements suggest a Gaussian distribution of copper nanoparticle sizes. However, due to the instability of Cu nanoparticles under a focused 200 kV electron beam, TEM characterization remains challenging. Nevertheless, the combined results from AFM and HR-TEM support a reliable estimate of the average particle size at approximately 1.2 nm.

Part II. Reflection spectroscopy.

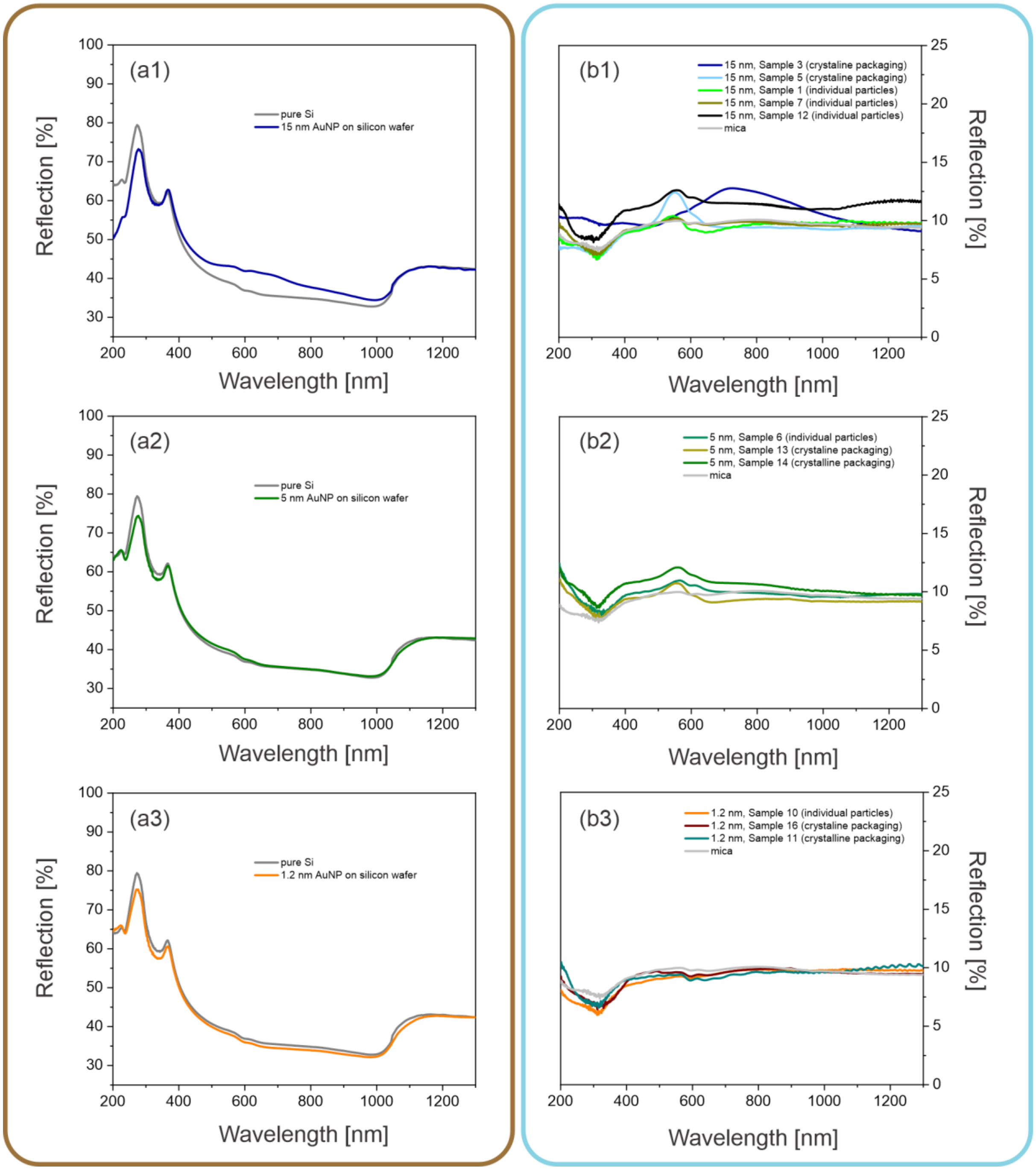

Figure SF3. Reflection spectrum of various samples on silicon (a, left) and mica (b, right) substrates using 15 nm (a1, b1), 5 nm (a2, b2) and 1.2 nm (a3, b3) particles in crystalline packaging and individually deposited on substrates.

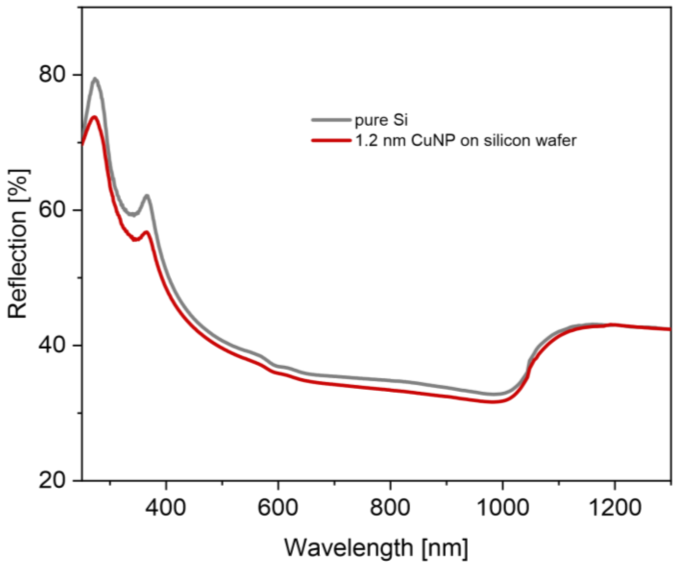

Figure SF4. Reflection spectra of a pristine silicon wafer (grey) and a silicon wafer decorated with 1.2 nm Cu nanoparticles (red). Notably, no plasmonic resonances are observed, indicating that a single layer of Cu nanoparticles of such size does not introduce detectable optical resonances in this spectral range.

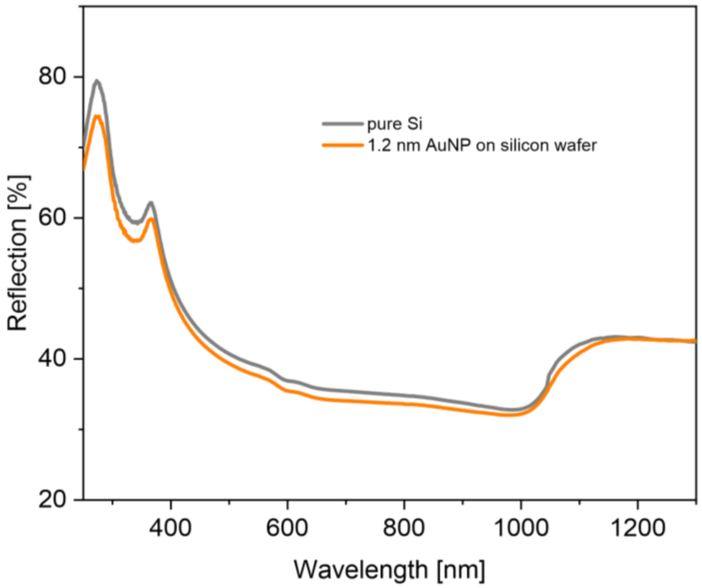

Figure SF5. Reflection spectra of a pristine silicon wafer (grey) and a silicon wafer decorated with 1.2 nm Au nanoparticles (orange) (data presented in Figure SF3a3). Notably, no plasmonic resonances are observed.

The reflection spectra of various samples, prepared by depositing Au nanoparticles of different sizes onto silicon wafers and mica, are shown in Figures SF3. 15 nm Au nanoparticles deposited on both substrates exhibit distinct plasmonic resonances around 600 nm with a significant deviation of peak position and spectral width from sample to sample (Figure SF1, a1 and b1). In contrast, samples coated with 5 nm nanoparticles display significantly weaker resonances with significantly less noticeable peak deviations (Figure SF1, a2 and b2). For samples coated with 1.2 nm Au nanoparticles, no discernible resonances are observed (Figure SF2, a3 and b3, Figure SF5), indicating the absence of plasmonically active metal structures measured on the macroscale in these samples. Similarly, a monolayer of 1.2 nm Cu nanoparticles does not exhibit any observable plasmonic or structural resonances, with the reflection spectrum closely following that of a clean, bare silicon wafer (Figure SF4 and Figure SF5).

Reflection spectra were measured on a Cary-7000 universal measurement spectrometer (Agilent, US). The spectral bandwidth used for all measurements is 1 nm.

Part III. Microscopic characterizations of decorated wafers

Figure SF6 presents additional data for 15 nm and 1.2 nm gold nanoparticles deposited on a bare silicon wafer. Bright-field imaging reveals large gold structures and agglomerates on the wafer surface (Figure SF6a2) when 15 nm particles are used. In contrast, the 1.2 nm nanoparticles are not visible under the same imaging conditions (Figure SF6b2). The scattering from these structures is negligible, suggesting a near-monolayer arrangement on the surface, as further confirmed by AFM mapping. Photoluminescence (PL) maps, however, display the opposite trend: the emission signals are more than an order of magnitude stronger for 1.2 nm samples (Figure SF6, a3 and b3). These signals scale inversely with particle size and consequently with the amount of gold, indicating that the emission originates from the Au-Si interface when the wafer is decorated with particles of an optimal size.

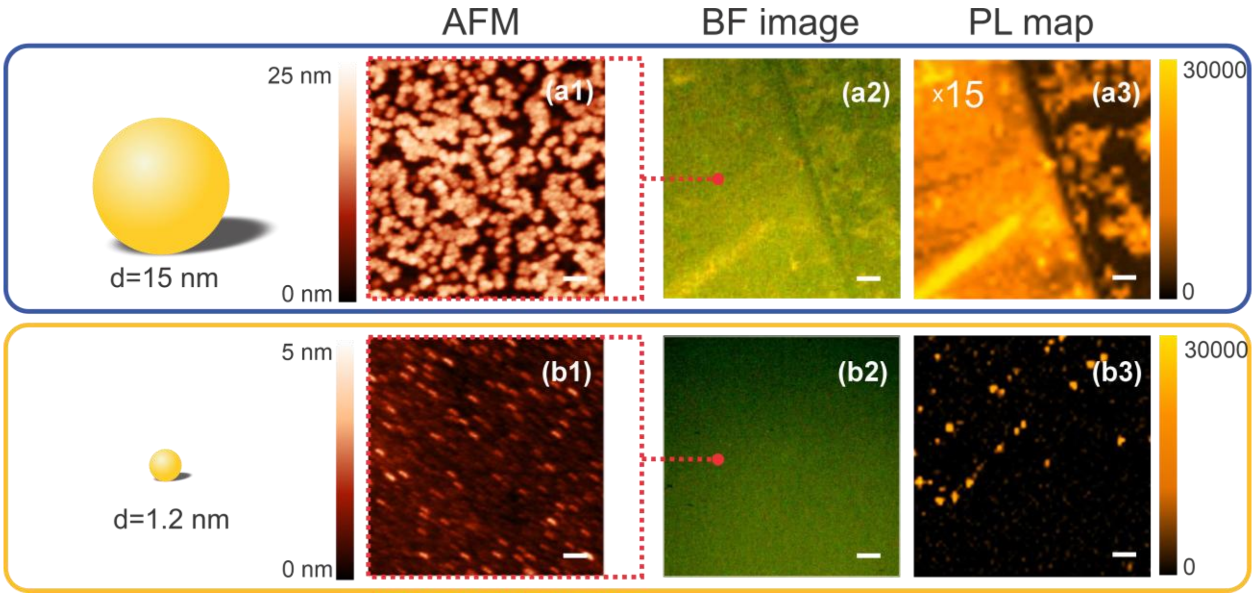

Figure SF6. Characterization and imaging of wafer surfaces decorated with 15 nm (a) and 1.2 nm (B) gold particles: (1) atomic macroscopy maps (Scale bar 100 nm), (2) bright-field optical images using white light illumination, (4) photoluminescence (PL) maps at 630 nm. Scale bar 5 μm for all optical images. Note: PL maps at 630 nm for 15 nm (a3) are enhanced by a factor of 15.

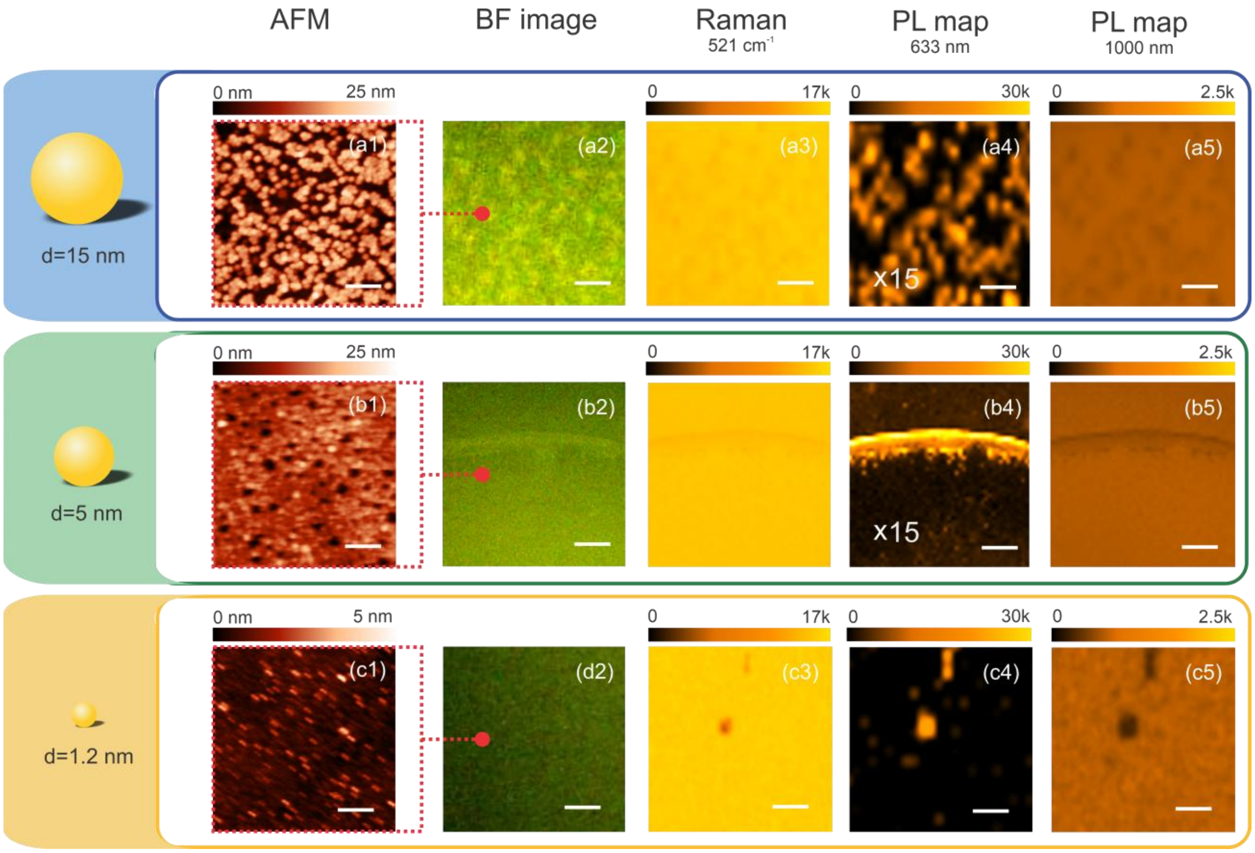

Figure SF7. Characterization and imaging of wafer surfaces decorated with 15 nm (a), 5 nm (b) and 1.2 nm (c) Au particles: (1) atomic microscopy maps (Scale bar 200 nm), (2) bright-field optical images using white light illumination, (3) Raman maps at the 521 cm^{-1} silicon phonon line, (4) photoluminescence (PL) maps at 630 nm, (5) PL maps at 1000 nm, representing silicon's phonon-assisted luminescence from the bottom of the conduction band at X point. Scale bar 5 μm for all optical images. Note: PL maps at 630 nm for 15 nm (a4) and 5 nm (b4) samples are enhanced by a factor of 15.

Similar to Figure 2 of the main manuscript, Figure SF7 provides detailed AFM maps and spectral images of samples with nanoparticles of varying sizes. While bright-field white light images reveal light-scattering structures for the larger particles, the 1.2 nm particles do not exhibit any discernible contrast under white light illumination. On the other hand, the PL image (Figure SF7c4) is significantly brighter for the 1.2 nm particles compared to the larger particles (Figures SF7a4 and SF7b4). Importantly, the Raman signals and the phonon-assisted PL near the bottom of the conduction band, both intrinsic emission signatures of Si, are significantly suppressed for the 1.2 nm particles. These signals remain unaffected in the case of the 15 nm and 5 nm particles, which are clearly visible in bright-field reflection/scattering.

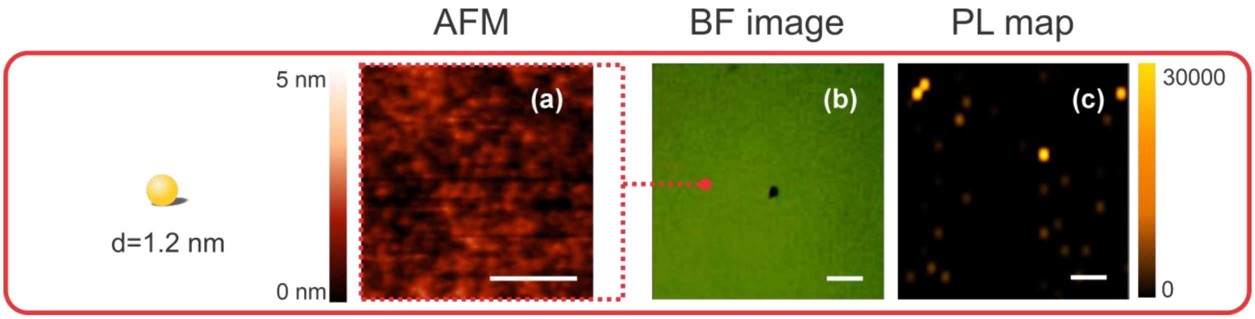

Figure SF8. Characterization and imaging of wafer surfaces decorated with 1.2 nm Cu particles: (1) atomic force microscopy maps (Scale bar 400 nm), (2) bright-field optical images using white light illumination, (4) photoluminescence (PL) maps at 630 nm. Scale bar 5 μm for all optical images.

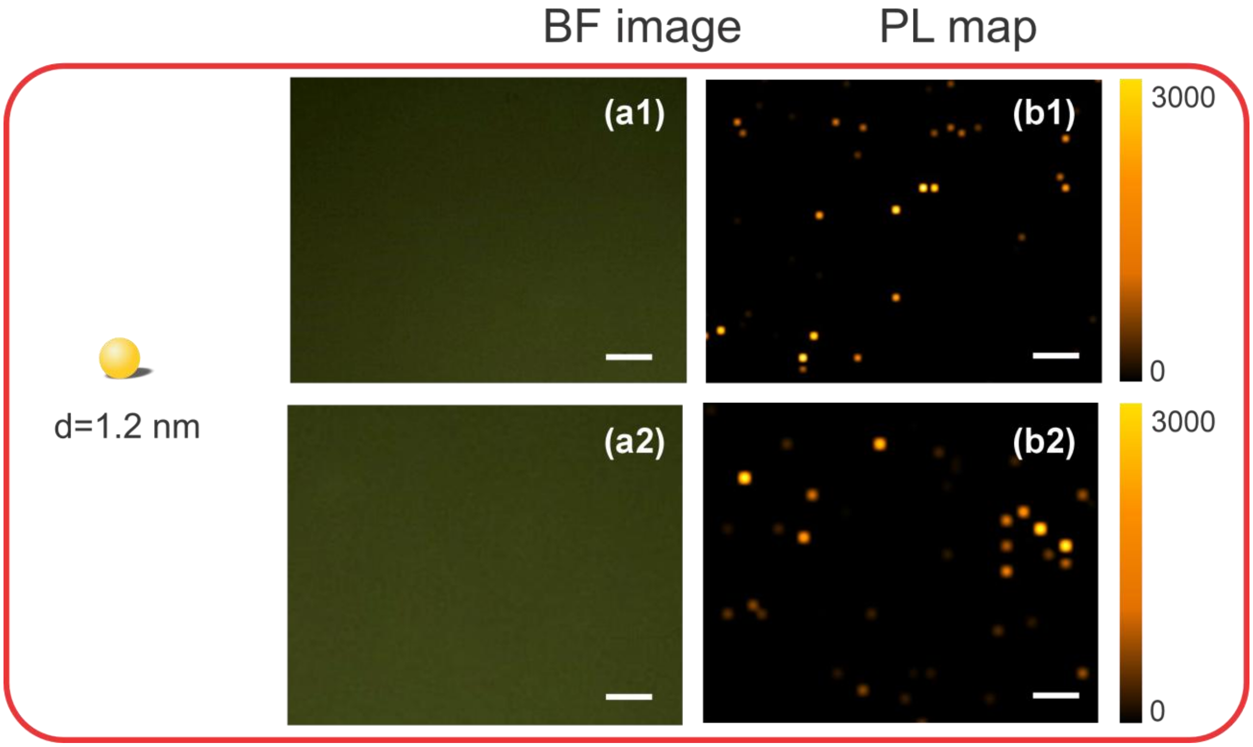

Figure SF9. Characterization and imaging of wafer surfaces decorated with 1.2 nm Cu particles: (a1, a2) bright-field optical images using white light illumination, (b1, b2) photoluminescence (PL) maps at 630 nm. Scale bar 10 μm for all images.

Two key observed experimental correlations:

1. The photoluminescence maps associated with momentum-enabled emission in silicon using 1.2 nm Au (Figure 2d4, Figures SF6b3, Figure SF7c4) and 1.2 nm Cu (Figure SF8c, Figure SF9b1, Figure SF9b2) exhibit bright emission in regions where the optical images show no discernible surface features. This contrasts sharply with the 5 nm and 15 nm samples, where visible scattering marks the presence of larger clusters (Figure 2b4, Figure 2c4, Figure SF6a3, Figure SF7a4, Figure SFb4).

2. The spatial distribution of PL intensity is inversely correlated with the characteristic spectroscopic responses of silicon, including the Raman line (Figure 2d3, Figure SF7c3) and the phonon-assisted band-edge emission (Figure 2d5, Figure SF7c5). Such anticorrelation is consistent with the suppression of phonon-assisted recombination and the activation of phononless electronic transitions, in line with the formation of momentum-expanded photonic states.

The non-uniform, "patchy" nature of the PL emission from bulk silicon decorated with 1.2 Au (Figure 2d4, Figures SF6b3, Figure SF7c4) and 1.2 nm Cu (Figure SF8c, Figure SF9b1, Figure SF9b2) nanoparticles likely reflects the highly specific spatial and structural requirements for this process to occur. Several conditions must coincide: multiple nanoparticles of the appropriate size must reside within an optimal nanoscale distance from the silicon surface and exhibit inter-particle distances that sustain a sufficiently high density of carriers in the silicon material near the interfacial region. Because this light–matter interaction is extremely short-ranged, even minimal variations in the nanoparticle–surface separation (e.g., due to an oxide layer) or inter-particle separation can suppress the effect, resulting in the observed non-uniform emission pattern.

The temperature of bulk silicon near the interface can be determined using Raman thermometry discussed in [3,4] and derived from the spectral shift of the Si Raman line. Calibration between the Raman peak position and temperature used a known coefficient of -0.022 cm^{-1}/K, enabling temperature estimation with an accuracy of ±5 K.

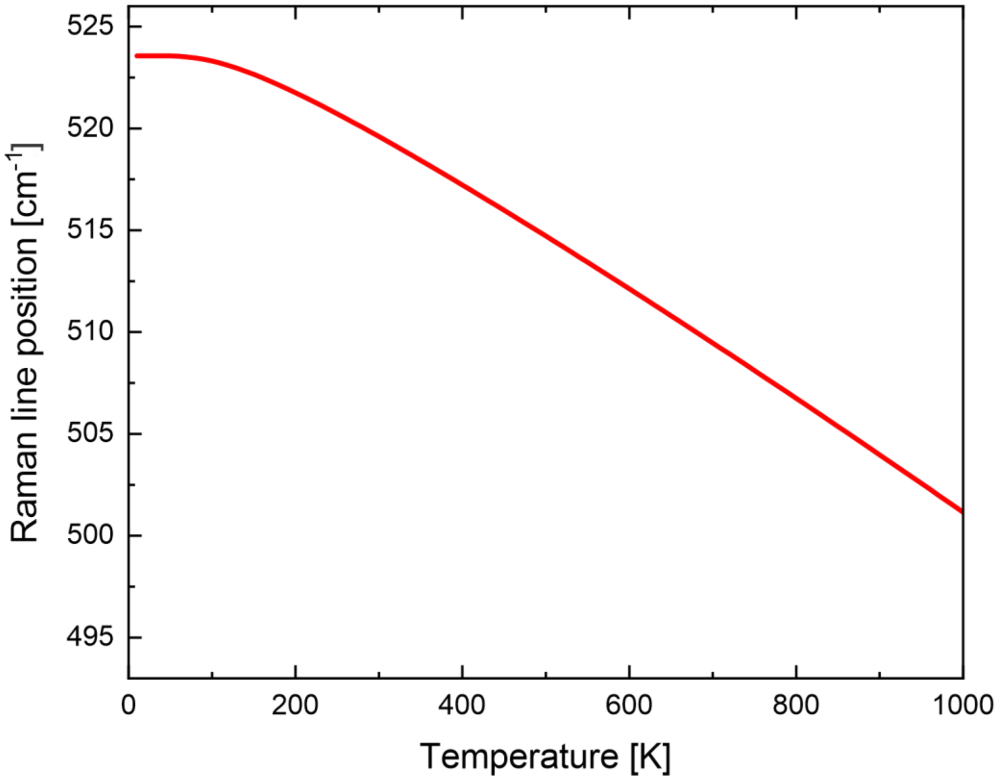

Figure SF10. Bulk Si Raman line spectral peak shift as a function of temperature.

We note that, in contrast to the localized high-temperature conditions reported in [3] for tip-based geometries, the present planar bulk configuration does not exhibit measurable Raman peak shifts or line broadening (Figure 3a) across all samples decorated with nanoparticles of varying sizes, indicating that the silicon remains near room temperature under our experimental conditions.

All micro-spectroscopy experiments were performed on a custom-modified microscopy system based on an InVia Renishaw system using 532 nm and 785 nm laser sources. The samples were illuminated with a 0.75 NA air objective (Leica), and emission was collected in the epi-configuration. The spectra were measured in expanded mode with a 1200 gr/mm diffraction grating used for spectral scanning with 0.6 cm^{-1} resolution over the entire spectral region. The spectrometer is equipped with a spectrally calibrated Centrus™ detector, a thermoelectrically cooled CCD detector proprietary to Renishaw Inc. All emission spectra are corrected for the spectral response of the detection system.

Part IV. Spectral and temporal characterization and comparison.

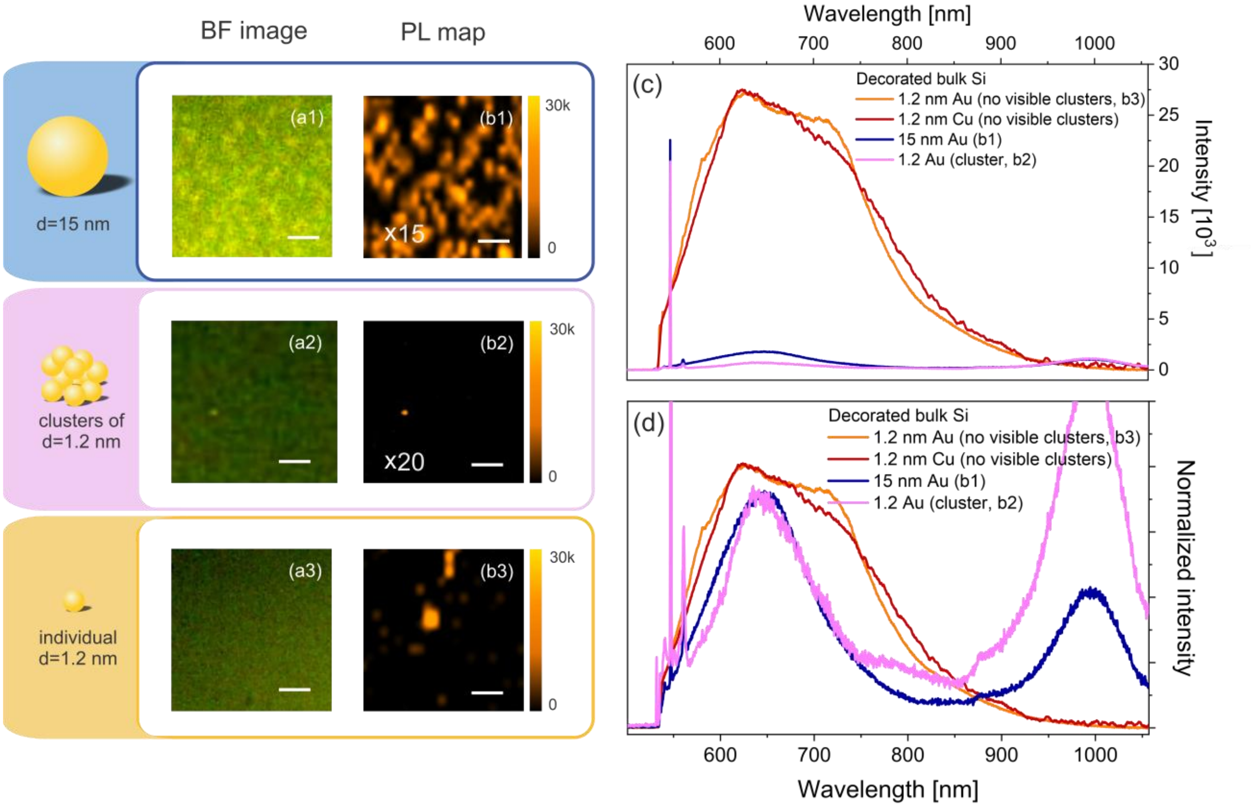

Figure SF11. Characterization and spectral imaging of silicon wafer surfaces decorated with 15 nm and 1.2 nm gold nanoparticles. (a1, b1) Optical and PL map of surfaces coated with 15 nm Au nanoparticles and with 1.2 nm Au nanoparticles, respectively. Panels (a2, b2) correspond to regions containing optically visible clusters, while (a3, b3) show areas of optically clean topography, where 1.2 nm Au nanoparticles form an almost continuous single layer on the silicon surface. (c) Relative emission spectra from all samples, acquired under identical experimental conditions (Excitation using 532 nm, 0.5 mW, 0.75 NA). (d) Normalized spectra reveal that 15 nm Au NP or large clusters comprised of 1.2 nm particles exhibit narrow and stable emission, whereas a uniform layer of individual 1.2 nm Au nanoparticles on the surface of bulk silicon produces an ultrabroad and intense emission, distinctly different from the conventional photoluminescence of gold.

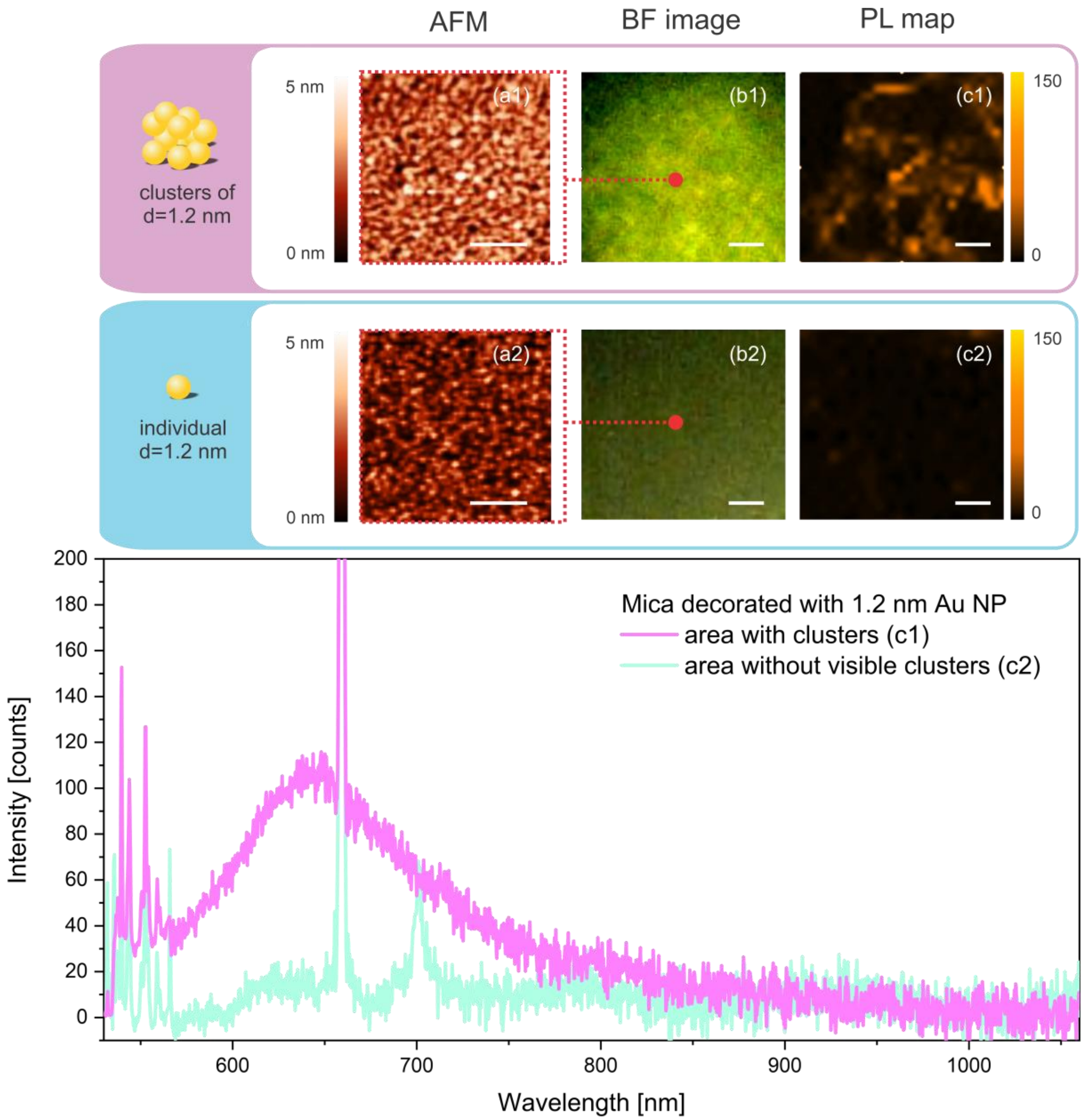

Figure SF12. Characterization and spectral imaging of mica surfaces decorated with 1.2 nm Au nanoparticles. (a1, b1, c1) AFM, optical, and PL maps of regions containing optically visible gold deposits and clusters composed of 1.2 nm Au nanoparticles. (a2, b2, c2) Corresponding characterization of an optically smooth region where the 1.2 nm Au nanoparticles form an almost continuous single layer on the mica surface. (d) Relative emission spectra from both the clustered and single-layer regions on mica, acquired under identical experimental conditions (excitation at 532 nm, 0.5 mW, 0.75 NA objective).

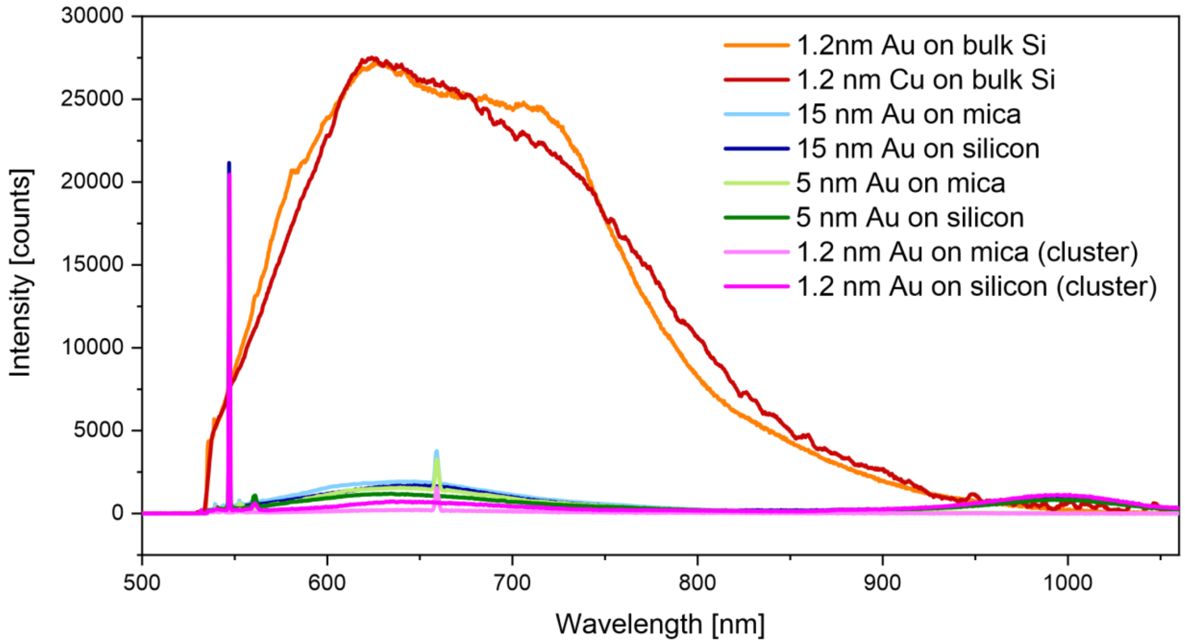

Figure SF13. Emission spectra of Au and Cu nanoparticles deposited on different surfaces. The spectra clearly demonstrate that the emission intensity from 1.2 nm single-layer Au and Cu nanoparticles is significantly higher than that of intrinsic metallic photoluminescence. Moreover, when 1.2 nm Au or Cu nanoparticles are introduced onto the silicon surface, the resulting emission spectra are virtually identical in both shape and intensity. Excitation conditions: 532 nm, 0.5 mW, 0.75 NA objective.

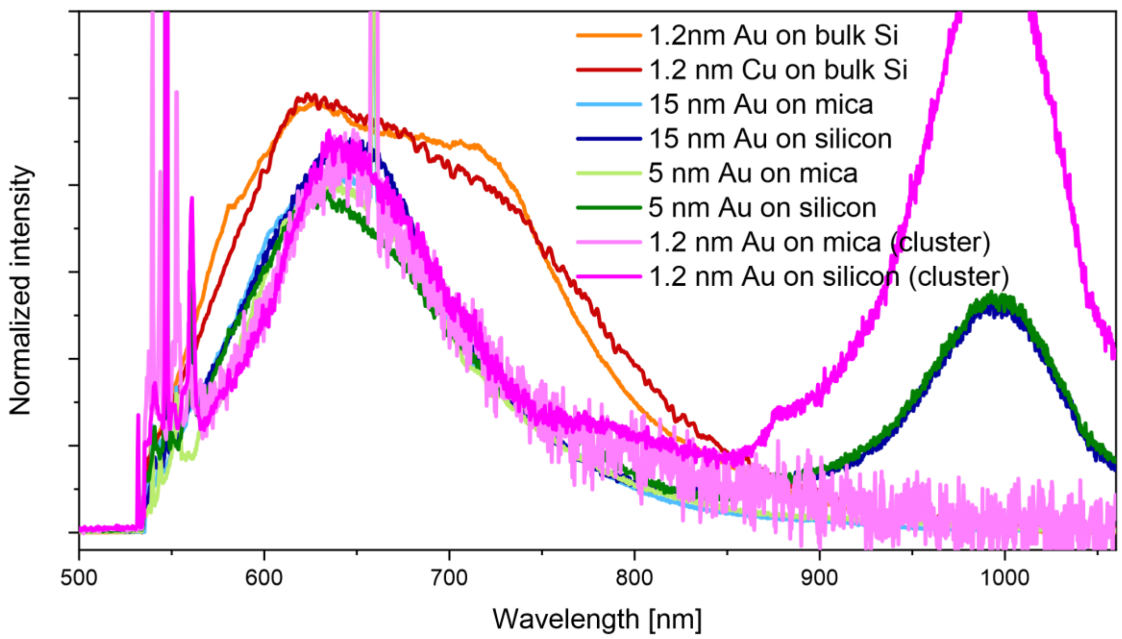

Figure SF14. Normalized emission spectra of Au and Cu nanoparticles deposited on different surfaces, corresponding to the samples shown in Figure SF13. The spectral widths of the emission from large nanoparticles or from clusters composed of 1.2 nm Au nanoparticles are consistent with the characteristic photoluminescence of bulk metals (Δ~0.3 eV). In contrast, when 1.2 nm Au or Cu nanoparticles are introduced onto the bulk silicon surface, the resulting emission becomes significantly broader (Δ~0.62 eV), indicating a distinct emission mechanism compared to intrinsic metallic photoluminescence.

The newly opened radiative channel effectively renders the transition direct (phononless), substantially increasing the transition probability. As a result, the radiative rate accelerates by more than three orders of magnitude relative to conventional silicon, with photoluminescence (PL) lifetimes reduced from the typical microsecond range to the nanosecond regime. Figure SF15 presents the time-resolved PL data for 1.2 nm Au and 1.2 nm Cu particles on a clean silicon wafer. Both cases yield lifetimes of approximately ~2 ns. In the context of our model, this indicates that the decay dynamics are fully dominated by the newly opened radiative channel, while the intrinsic microsecond non-radiative pathways of silicon remain unchanged. These time-resolved PL measurements also corroborate the material-agnostic nature of the light confiners. When either 1.2 nm Cu or 1.2 nm Au clusters are placed in close proximity to the silicon surface, the resulting emission exhibits indistinguishable spectral shapes in both the frequency domain (Figure 2 of the main manuscript) and the time domain (Figure SF15). This identical behavior provides strong evidence that the emission mechanism is independent of the confining material and must therefore originate from silicon itself.

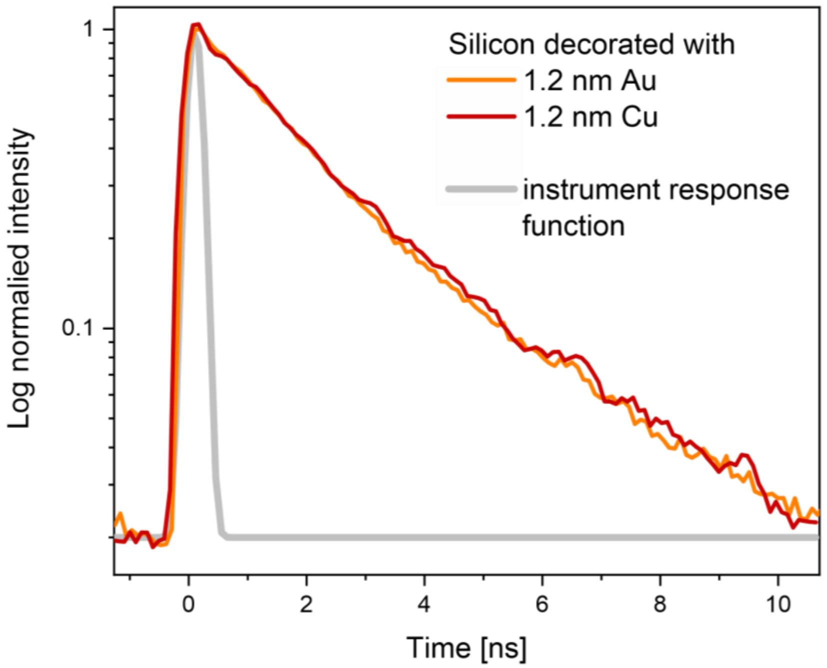

Figure SF15. Normalized time-resolved dynamics of the emission from a silicon surface decorated with 1.2 nm Au (orange) and 1.2 nm Cu (red) nanoparticles. Both traces exhibit virtually identical lifetimes of ~2 ns, consistent with expectations for phononless (direct) radiative transitions, analogous to allowed transitions in direct-bandgap semiconductors.

Time-resolved photoluminescence measurements has been conducted using Leica SP8 Falcon system, a customized commercial imaging platform, equipped with white-light picosecond laser source, which consists of a high-energy pulsed IR-fiber laser that is fed through the photonics crystal fiber to generate a spectral continuum. The fluorescence lifetime expeirments were conducted in epi-geometry using 10x objective, 514 nm excitation and signals detected using FALCON FLIM detector (Leica). Time resolution 120 ps.

Part V. Emission from Au nanoclusters.

The earliest studies of photoluminescence (PL) from bulk gold and other noble metals date back several decades, including the pioneering works by Mooradian [5]. The optical properties of bulk gold, including absorption and emission, are predominantly governed by interband transitions between the sp-hybridized band near the Fermi level and d-band states. While the emission spectra of gold nanostructures or nanostructured surfaces, including rough metal films, share some similarities with bulk gold, they also exhibit distinct spectroscopic features. These features include variable spectral shapes and peak positions, accompanied by enhancement mechanisms that remain the subject of active debate. Research has primarily focused on nanostructures supporting plasmonic resonances, such as nanospheres [6,7], rough metal surfaces [8,9], nanorods [10-14], bipyramids [15], nanocubes [16], and nanoshells [17]. Many studies attribute the broad Stokes emission spectrum to electronic Raman scattering (ERS) [18-21] or, alternatively, intraband transitions within the *sp* band [22,23]. Both mechanisms are typically forbidden in bulk gold due to the mismatch in momentum between the initial and final electronic states, which cannot be mediated by a free-space photon. Intraband transitions are also dipole-forbidden due to the identical symmetry of the involved electronic states. However, all constraints can be circumvented when the photon is strongly confined, enabling both the necessary momentum compensation and allowing higher-order transitions

To identify the physical mechanisms responsible for the photo-induced emission associated with the metal particles in our study, we prepared a series of samples consisting of Au nanoparticles of varying sizes, deposited on silicon and mica substrates. (see Methods and *Supplementary Information Part I*). Figure SF16 presents atomic force microscopy (AFM) images of 15 nm nanoparticles. These deposition methods resulted in two distinct morphologies: crystal-like structures (Figure SF16a, top row) and densely packed, yet individual particles on mica surfaces (Figure SF16a, bottom row). AFM characterizations of 5 nm and 1.2 nm Au nanoparticles deposited on mica are shown as well in Figure SF16 (b and c). As detailed in *Supplementary Information Part II*, samples with 15 nm and 5 nm nanoparticles exhibit weak plasmonic resonances to varying degrees. In contrast, 1.2 nm nanoparticles do not display plasmonic spectral features when measured in reflection mode at macroscopic scales (Figures SF3a3, measured with a 1 mm beam size).

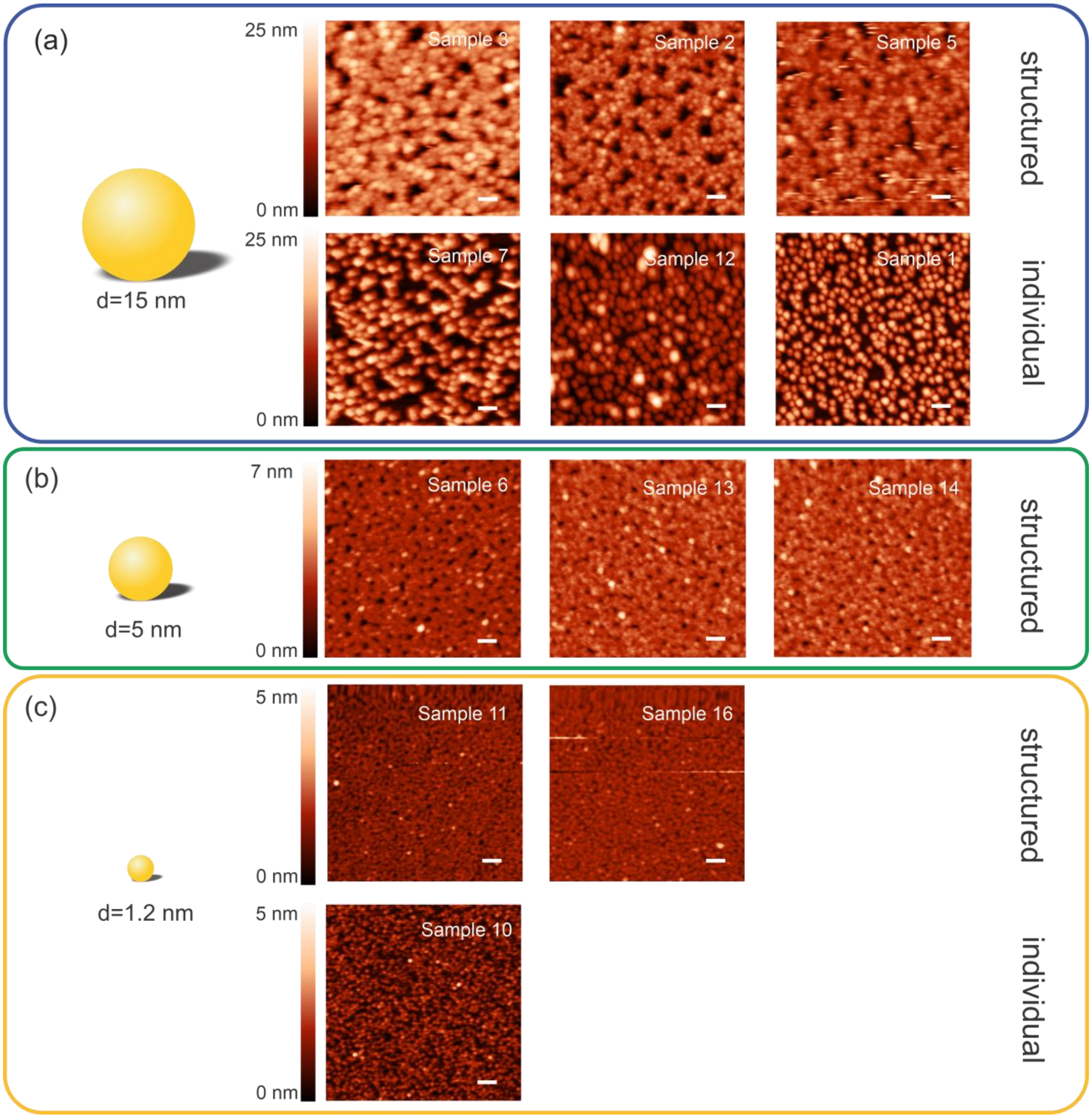

Figure SF16. Atomic force microscopy images of samples with 15 nm (a), 5 nm (b), and 1.2 nm (c) particles deposited on clean mica. Scale bar 100 nm. On samples 3, 2, and 5 (15 nm), as well as 11 and 16 (1.2 nm), the deposition resulted in particles forming a crystal-like order due to the specific deposition process described in *Supplementary Information Part I*. The following can be clearly seen in Figure SF17.

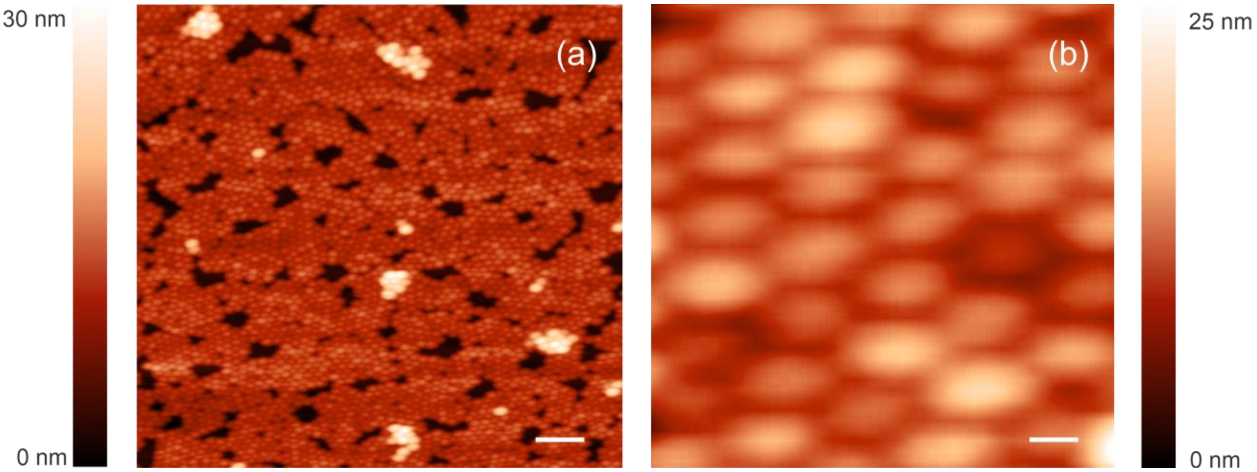

Figure SF17. Atomic force microscopy images of 15 nm particles deposited on clean mica. Scale bar 50 nm (a) and 10 nm (b). The images show a strong structural arrangement on the surface.

An example of the emission spectrum for 5 nm Au particles deposited on mica and silicon is shown in Figure SF18a. While the Raman features of the substrates remain independent of the excitation wavelength, emission from Au nanoparticles is observed only under 532 nm excitation and not with 785 nm. This confirms the PL origin of the emission, as supported by prior studies. [5,6,10,12-15,22] Weak phonon-assisted PL emission around 1000 nm for silicon-based samples is also visible under both 532 nm and 785 nm excitation, as expected and discussed in the main manuscript.

The PL signals arise from the energy separation between the d-band and sp-band near the Fermi surface, along with the joint density of states involved in these transitions. This energy separation varies across the Brillouin zone, with values of approximately 2.3 eV at the L-point and 1.35 eV near the X-point.[19] The energy band diagram shown in Figure SF18b, adapted from [24], incorporates both relativistic effects and spin-orbit coupling, as opposed to the simplified model used in reference [22]. Following the partial density of states, there should be a large population of electrons at the *d*-band that can be excited by a 532 nm photon (2.31 eV) to the *sp*-band at or above the Fermi level, where states are unoccupied. Excited electrons can then recombine with photo-created *d*-band holes, resulting in the emission of light. However, the signals that appear as the red tail of the visible luminescence warrant careful consideration, as thoroughly discussed in reference [22]. Thermalization of hot electrons in the conduction band makes them progressively more separated in momentum space from the region where holes are generated in the *d*-band (Figure

SF18b). Combined with a decrease in the density of states for these energies, this separation means that the red tail emission cannot be efficiently produced by conventional interband PL using free-space photons. Several emission mechanisms are plausible, all leveraging the confined photon's ability to carry significant momentum, thereby enabling momentum-forbidden diagonal transitions. One efficient pathway is a diagonal interband transition back to the d-band, as illustrated in Figure SF18b. For highly confined photons, this mechanism can easily extend the emission spectrum tail down to ~1.35 eV (~900 nm). Another plausible mechanism involves ERS or intraband transitions within the *sp*-band. Following a Fermi-Dirac distribution, electrons contributing to ERS must originate from states close to the Fermi level at room temperature. The steep dispersion of the *sp*-band should enable an ultrabroad (~1 eV), continuous emission on the Stokes side of the Rayleigh line, while anti-Stokes emission should be significantly suppressed due to the full occupation of states below the Fermi level. Strong experimental evidence of the ERS/intraband nature of the redtail emission is provided in reference [22]. Using an excitation photon energy below the *d*-to-*sp* band separation threshold, the authors observed a quadratic dependence of the emission on incoming flux at the center of the visible PL spectrum, consistent with the power law expected for a two-photon process. In contrast, the far-red tail exhibited a linear dependence. Since single-photon excitation cannot create a *d*-band hole in this regime, the emission must originate from *sp*-band electrons near the Fermi level. This finding strongly supports distinct origins for the emission at the center of the visible spectrum (conventional PL) and its far-red tail (diagonal ERS/intraband). In our experiments employing a single-photon excitation scheme, all parts of the emission spectrum of Au particles deposited on mica exhibit a linear dependence on the incoming photon flux (Figure SF18c). Figure SF18d shows the relative (left) and normalized (right) emission spectra for 15 nm, 5 nm, and 1.2 nm Au nanoparticles on mica.

In sum, the comprehensive characterization - including power dependence, excitation photon energy dependence, and the influence of structural ordering - strongly indicates that the observed signals in the current experiments and material system are most likely due to photoluminescence (PL), rather than electronic Raman scattering (ERS), originating from the 1.2 nm particles.

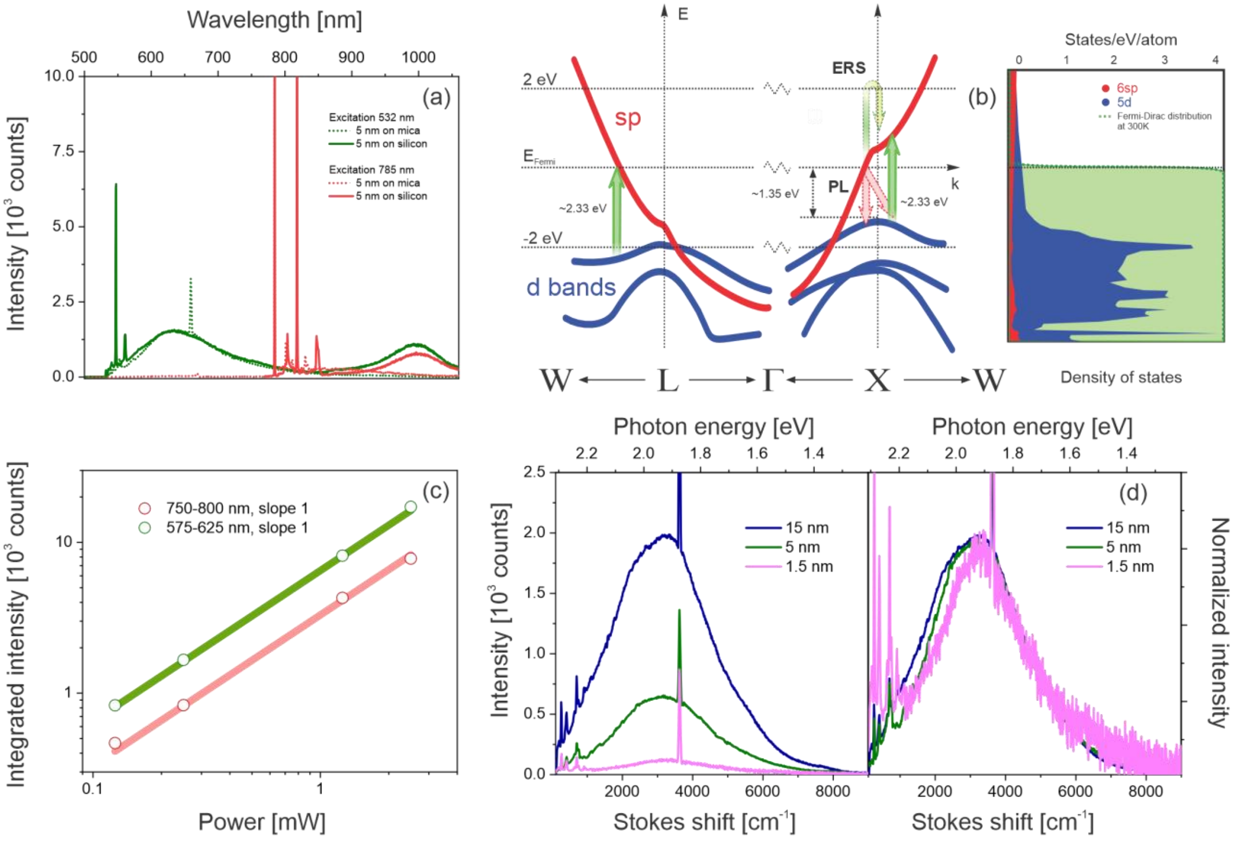

Figure SF18. (a) Emission spectra of 5 nm particles deposited on silicon (solid lines) and mica (dotted lines) under 532 nm (green lines) and 785 nm (red lines) excitation wavelengths. (b) Energy-momentum dispersion diagram for pure Au calculated including both relativistic effects and spin-orbit coupling.[24] The energy separation of 2.3 eV near the L point and 1.35 eV near the X point of the Brillouin zone allows effective excitation followed by interband emission. Right panel: partial density of states for 6sp and 5d bands. Density of states increases significantly ~2 eV below the Fermi level, with a maximum around 2.4 eV.[25] (c) Power dependence of signal from 5 nm Au particles integrated over different parts of the emission spectrum at PL (575-625 nm) and ERS/intraband (>750 nm) plotted on a log-log scale. Both dependencies can be fitted with a linear function of slope 1. (d) Relative (left) and normalized (right) emission spectra of Au particles of different sizes deposited on mica.

Part VI. Spectroscopic analysis of chemical ligands involved in the synthesis and deposition.

Fluorescence spectroscopy of batch solutions containing compounds used in synthesis and deposition was performed using a Shimadzu RF6000 fluorescence spectrometer. The emission properties of nicotinamide adenine dinucleotide (NAD) in solution, along with other compounds used in synthesis and deposition, are presented below. A highly concentrated NAD solution does not exhibit any visible emission under excitation at 310 nm or 530 nm (Figure SF19). When NAD is attached to 1.2 nm Au nanoparticles, its emission is modified, but only weak signals appear at 400 nm, even at high concentration (Figure SF20b), with no detectable emission at 530 nm (Figure SF20c). Finally, when all components, including Bis(p-sulfonatophenyl)phenylphosphine dihydrate dipotassium salt (BSPP), are present at high concentration, the emission spectrum under 310 nm excitation displays spectral signatures of all the compounds (Figure SF21b), while no detectable emission is observed under 530 nm excitation, the wavelength used in this study (Figure SF21c).

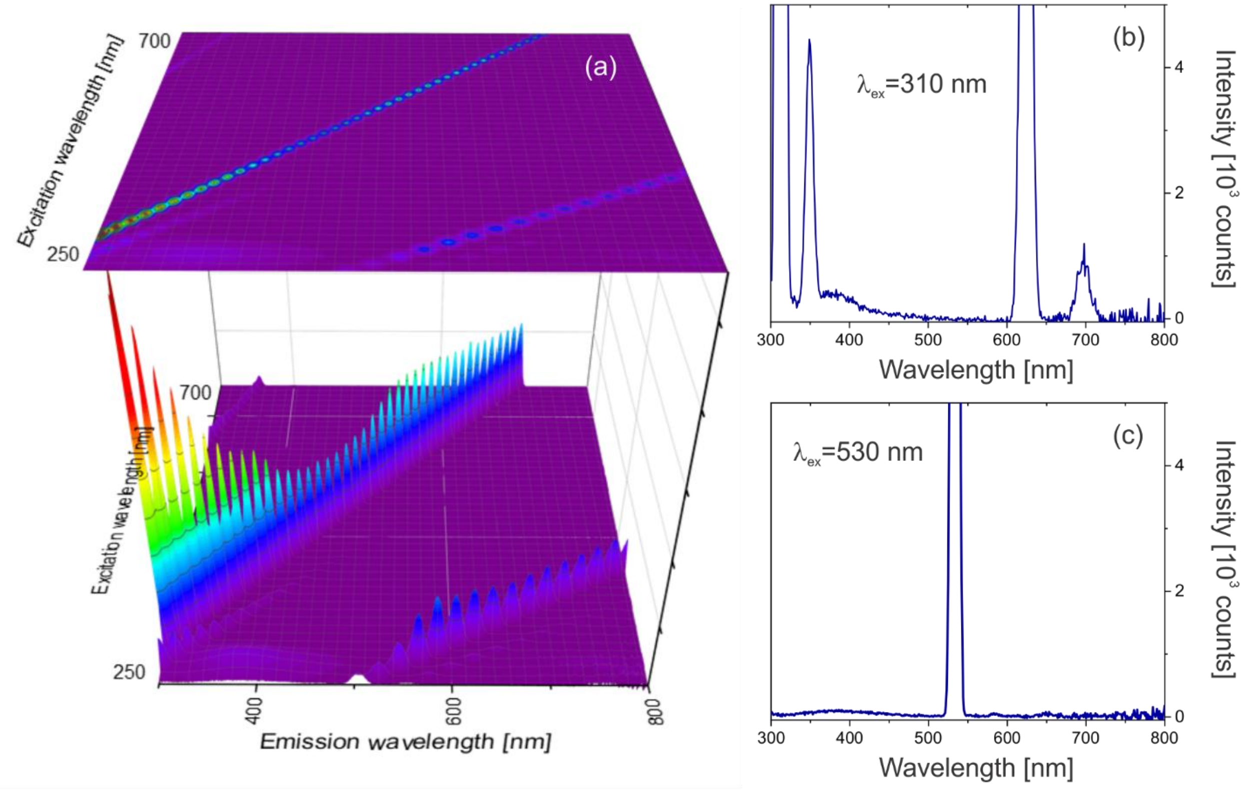

Figure SF19. Excitation-emission map of a highly concentrated pure NAD solution. No visible emission is detected when excited at its absorption band (b, 310 nm) or at the excitation wavelength used in the main study (c, 530 nm). The sharp emission bands correspond to the first- and second-order diffraction of the excitation wavelength.

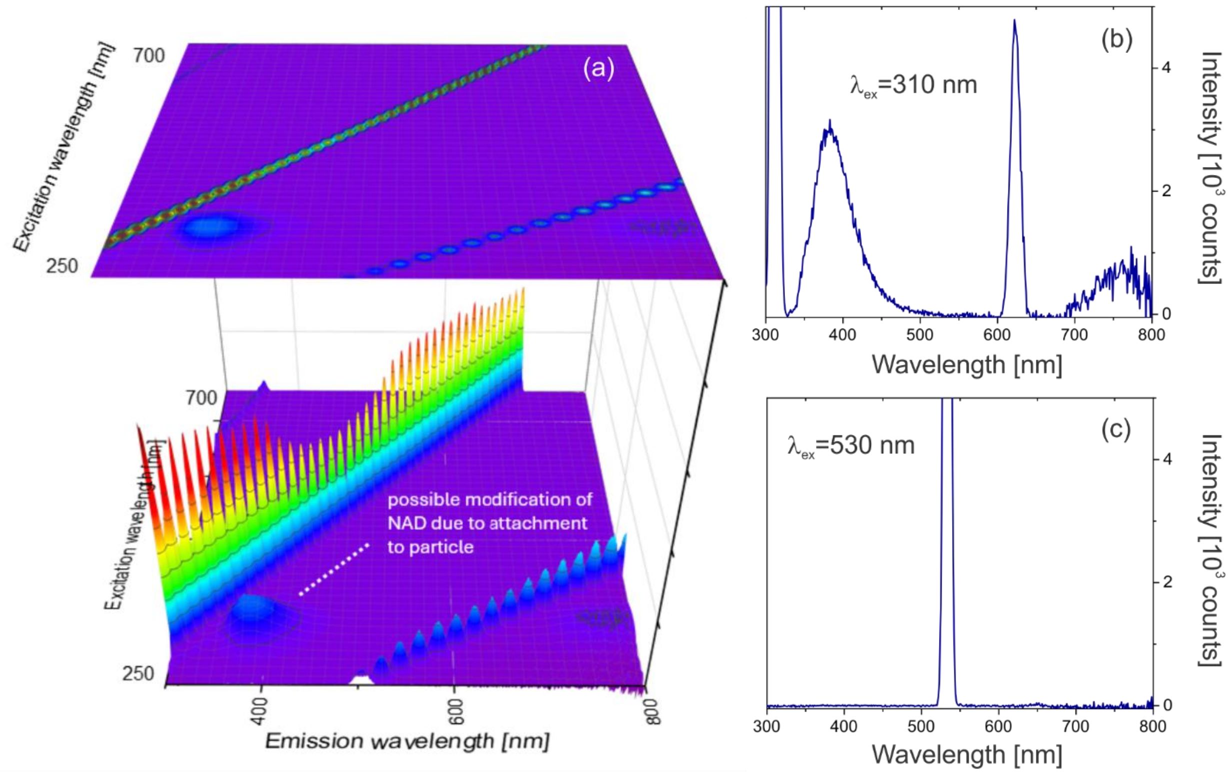

Figure SF20. Excitation-emission map of a highly concentrated solution of 1.2 nm Au nanoparticles with NAD (NAD-NPs). Weak emission at 400 nm appears when NAD is attached to the nanoparticles and excited at 310 nm. No visible emission is detected when excited at 530 nm, the excitation wavelength used in the main study. The sharp emission bands correspond to the first- and second-order diffraction of the excitation wavelength.

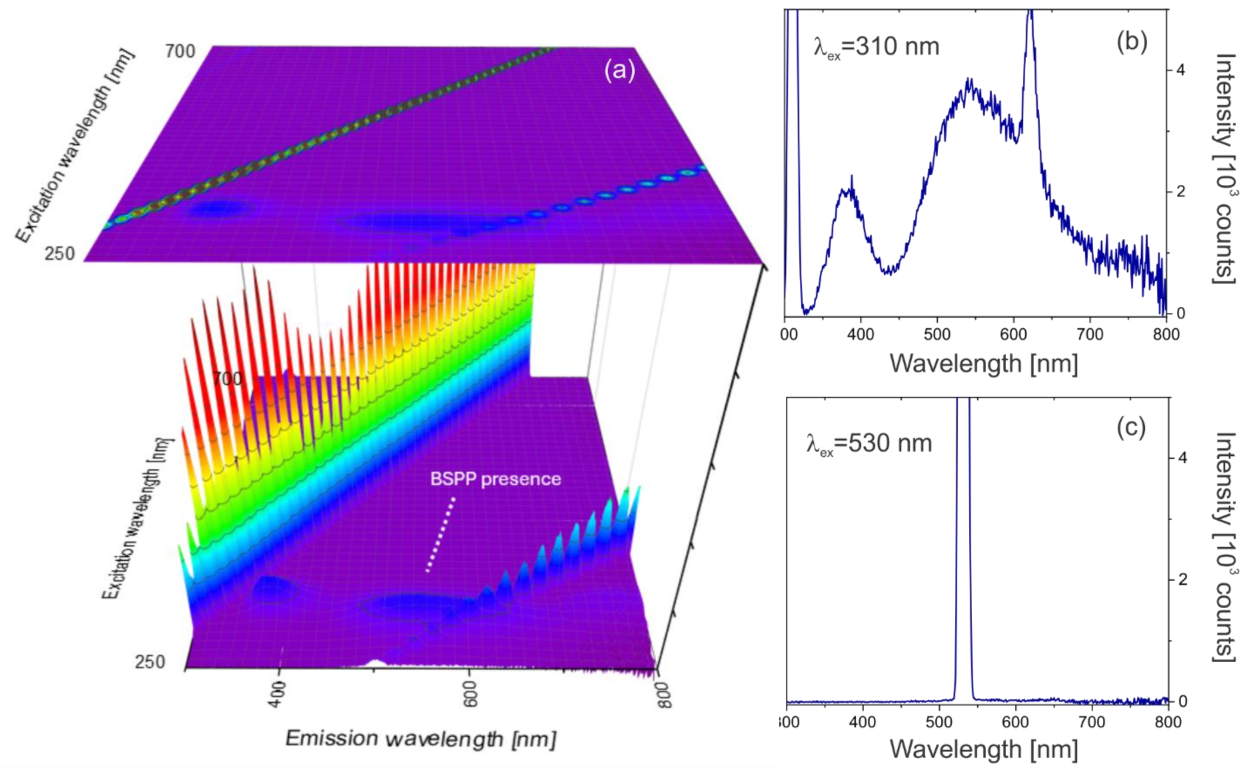

Figure SF21. Excitation-emission map of a highly concentrated solution of 1.2 nm Au nanoparticles with NAD and BSPP. The emission spectrum exhibits weak spectral signatures of all components when excited with 310 nm (b), and no detectable emission when 530 nm excitation is used. The sharp emission bands correspond to the first- and second-order diffraction of the excitation wavelength.

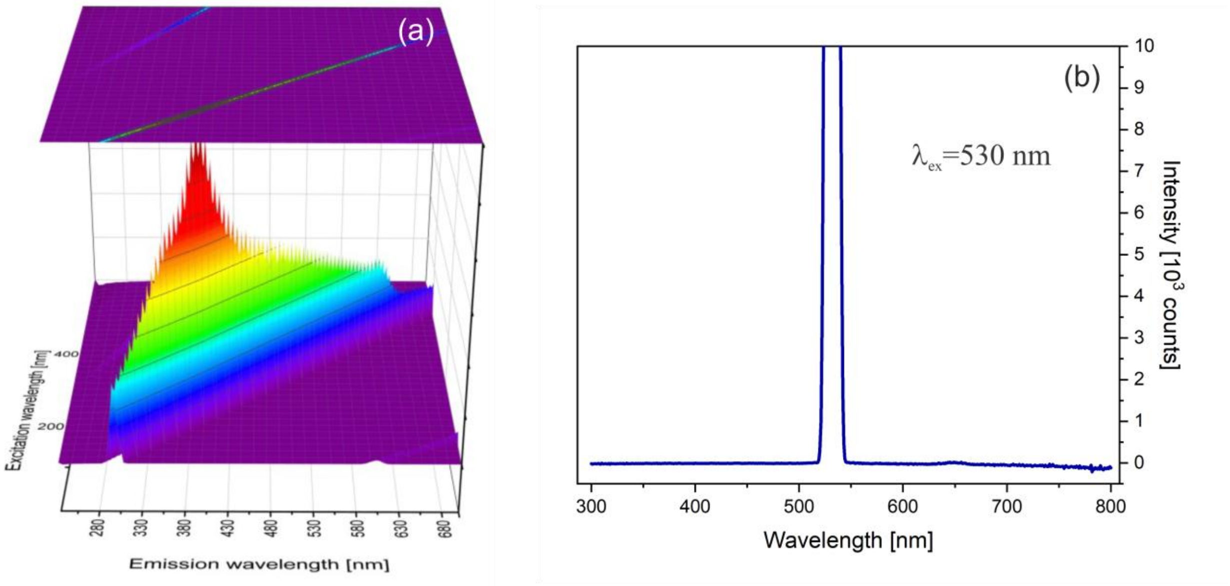

Figure SF22. Excitation-emission map of a highly concentrated solution of 1.2 nm Cu nanoparticles. The emission spectrum does not exhibit any detectable emission across a broad spectral range.

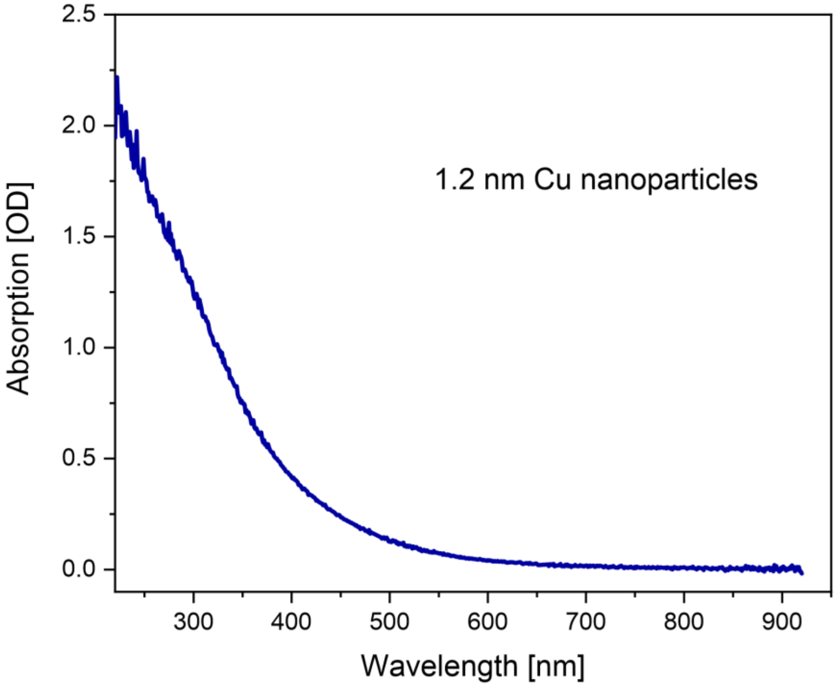

Figure SF23. Absorption spectrum of 1.2 nm Cu nanoparticles.

Part VII. Si coated with multiple layers of 1.2 nm particles.

Figure SF24 shows reflection spectra of pure silicon, silicon coated via sputtering of gold, as well as 1.2 nm particles forming a multi-layer crystal-like structure on the silicon wafer surface. First, when gold is sputtered on the silicon surface, it starts to reveal bulk metal properties. When working as a semitransparent mirror in near-IR, 7 nm and 10 nm coated Au films on Si demonstrate strong absorption above the plasma frequency (~400 nm) (Figure SF24, green and dark yellow spectra). Interestingly, the multi-layer arrangement of 1.2 nm Au particles does not reveal bulk gold properties. While the plasmonic resonance becomes visible, the film remains transparent. The spectrum displays not only the features of the indirect bandgap of silicon but also distinct absorption lines near and above the direct bandgap of the semiconductor substrate. When normalized at 1300 nm, the spectrum shows both an increase in absorption and the slope of indirect bandgap absorption. This observation strongly supports the notion of photon-momentum-enabled absorption in silicon. Overall, the multi-layer arrangement provides a transparent yet dense coating of the silicon surface. In Figure SF25a, the emission spectrum of the sample with a multi-layer film of 1.2 nm particles is plotted. Importantly, the emitted signal is of the same magnitude and exhibits the same trends observed for a monolayer film of 1.2 nm particles coated on the wafer surface. Moreover, the PL map measured at 650 nm reveals no correlation with gold film topography. As seen from Figure SF25 (b and c), the multi-layer film exhibits a significant variation in thickness and structure. At the same time, the PL maps remain flat, without any correlation with the visible gold topography. This observation further supports the notion that detected signals originate from the silicon-gold interfacial region and are independent of the gold layer's thickness.

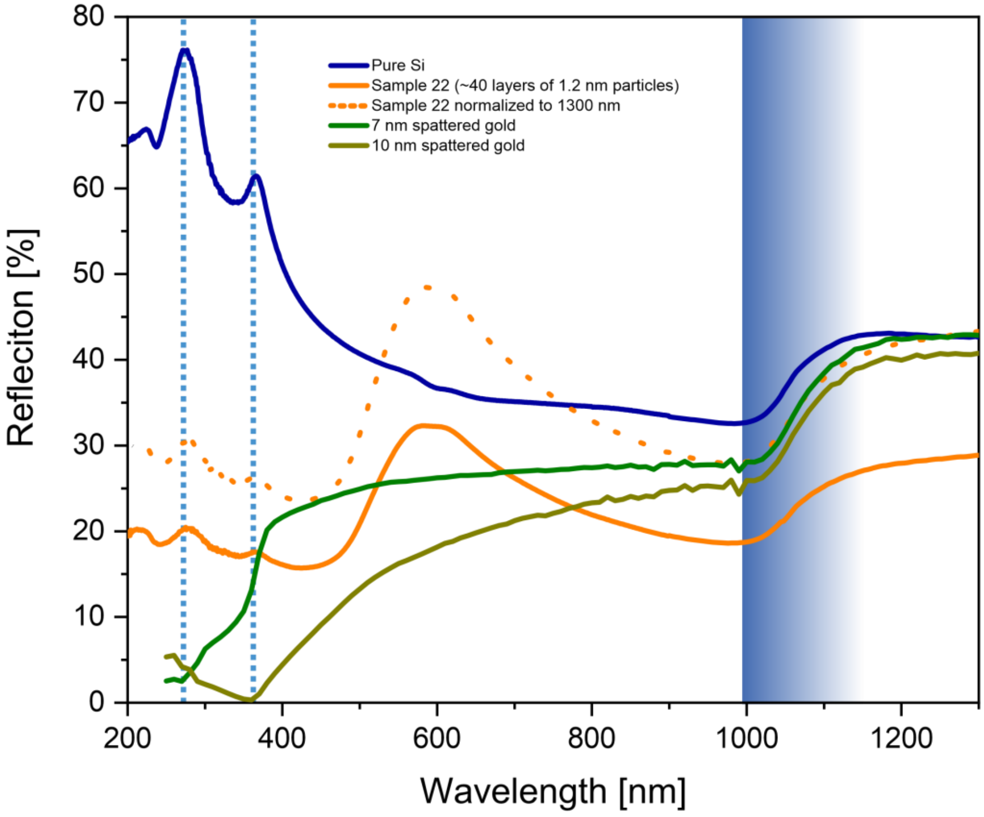

Figure SF24. Reflection spectra of pure silicon wafers and silicon wafers coated with gold. The coatings were applied either via sputtering or by depositing 1.2 nm gold particles to form a multi-layer film on the wafer surface. While the spectra of the sputtered gold coatings clearly exhibit distinct features characteristic of bulk gold, these features are absent in the thick, 65 nm multi-layer arrangement of 1.2 nm particles (~40 layers).

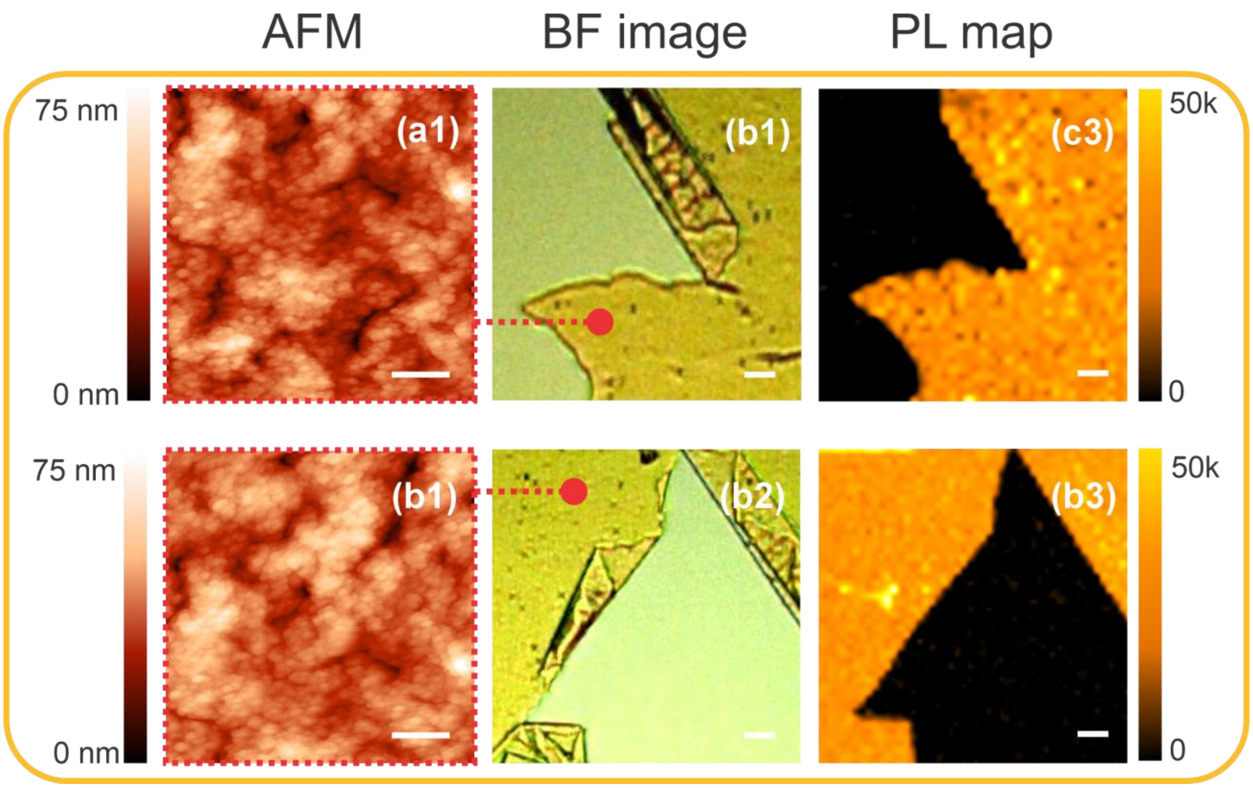

Figure SF25. (a1, b1) AFM mapping reveals a thick arrangement of ~40 layers of 1.2 nm gold particles. Scale bar 200 nm. (a2, b2) Bright-field images highlight variations in the layer's topography. (a3, b3) PL map acquired at 650 nm shows no correlation with the film's topography, indicating that the observed photoluminescence originates from the interfacial layer between the particles and the semiconductor substrate. Optical images a2-b2, a3-b3 scale bar 5 μm.

Part VIII. Confined photon (see also Supplementary Information to Ref [3])

While the energy of a photon is fixed by its angular frequency, $\hbar\omega$; outside vacuum, its momentum is tunable. In a dielectric, the canonical momentum of the photon increases $h/\lambda = nh/\lambda_0$ by the refractive index, n, which is a material property only in boundless media. Otherwise, as the imaginary counterpart of the absorption coefficient, it too is determined by the light-matter interaction, and may be gainfully defined by the extent of confinement of light, $n = \lambda_0/\lambda$. We note that the electromagnetic density in matter inseparably consists of Maxwell's displacement of electric field (light) and material polarization, $D = \varepsilon E = E + 4\pi P$, and $\varepsilon = n^2$, measures the extent to which the photon, as the quantized displacement, is light (field) $\varepsilon^{-1} = E/D$ or matter (polarization) $1 - \varepsilon^{-1}$. For nanometr-size confinement, $\varepsilon = (\lambda_0/\lambda)^2 \sim 10^4$, meaning the photon consists entirely of polarization $4\pi P/D = 1$. Photons can be atomically confined at plasmonic junctions [26,27], most famously in picocavities [28] of effective volume $V_e < (1\,nm)^3$ inferred from enhanced local fields, $E_L/E_0 \propto V_0/V_e$, as in the Purcell effect. However, V_e is not a physical volume, $V_e = \bar{\varepsilon} V_c$, where $V_c \ll V_e$ is the physical mode volume and $\bar{\varepsilon} = \langle \varepsilon(r,\omega) \rangle$ is the expectation value of the dielectric, which in general is nonlocal, complex and tensorial. Although it is nontrivial to associate a physical volume to a picocavity, it is clear that $V_c \ll V_e$ refers to the natural limit of atomic confinement. Photons can be directly seen to be atomically confined in tip-enhanced Raman microscopy, with measured Gaussian light-field distribution of standard deviation σ_x = 0.14 nm inferred from the TERS image resolution of FWHM = 0.16 nm [29,30]. As polarization, the confined photon is a time harmonic dipole, a quantum oscillator in operator notation $\hat{H}/_{\hbar\omega} = (a^\dagger a + 1/2)$ with a mode occupation number $n = \langle a^\dagger a \rangle = 1$, where $a^\dagger$and a are the creation and annihilation operators. For the photon in vacuum, q and p are the in-phase and quadrature components of the electric field, described by the conjugate quadrature operators, $\hat{q} = (a^\dagger + a)/2$ and $\hat{p} = i(a^\dagger - a)/2$ and with uncertainty relation $\sigma_q \sigma_p = (n + 1/2)$. For the polarization of the confined photon, as for a harmonic oscillator, they acquire their physical meaning of position and momentum.

To be more explicit, consider the dipolar polarization as the photon, with a wavefunction that takes the form of the n=1 state of the harmonic oscillator:

Photon wavefunction: $\psi(x) = N_x x e^{-\frac{x^2}{4s_x^2}}$

$$N_x = \left(\frac{1}{\sqrt{2\pi}s_x^3}\right)^{1/2}$$

Probability density: $|\psi(x)|^2 = N_x^2 x^2 e^{-\frac{x^2}{2s_x^2}}$

Reciprocal space: $\psi(k) = -iN_k k e^{-s_x^2 k^2} = -iN_k k e^{-\frac{k^2}{4s_k^2}}$

$$s_k = \frac{1}{2s_k},\ N_k = \left(\frac{\sqrt{8}s_x^3}{\sqrt{\pi}}\right)^{1/2} = \left(\frac{1}{\sqrt{2\pi}s_k^3}\right)^{1/2}$$

Probability density: $|\psi(k)|^2 = N_k^2 k^2 e^{-\frac{k^2}{2s_k^2}}$

Expectation values: $\langle x^2 \rangle = (2n+1)s_x^2 = \sigma_x^2$

$$\langle k^2 \rangle = (2n+1)s_k^2 = \sigma_k^2$$

Uncertainty principle: $\sigma_x \sigma_k = \left(n + \frac{1}{2}\right)$

Part IX. Highly degenerate electron gas.

Under our current illumination conditions, we calculate that for bare silicon, the carrier density in the conduction band is on the order of 10^{19} cm^{-3}. In the presence of confinement, our model stipulates that the transition probability in silicon is enhanced by about three orders of magnitude, which would give rise to a higher density of conduction electrons in the immediate vicinity of the confiner. Figure SF26 shows the calculated electron density in the conduction band, when the absorption rate near the interfacial region is enhanced by 100 times (red), 300 times (green), or 500 times (blue). At these densities, the electron gas is in the highly degenerate regime. Note that an electron density of 10^{21} cm^{-3} corresponds to one excited electron per nm^3.

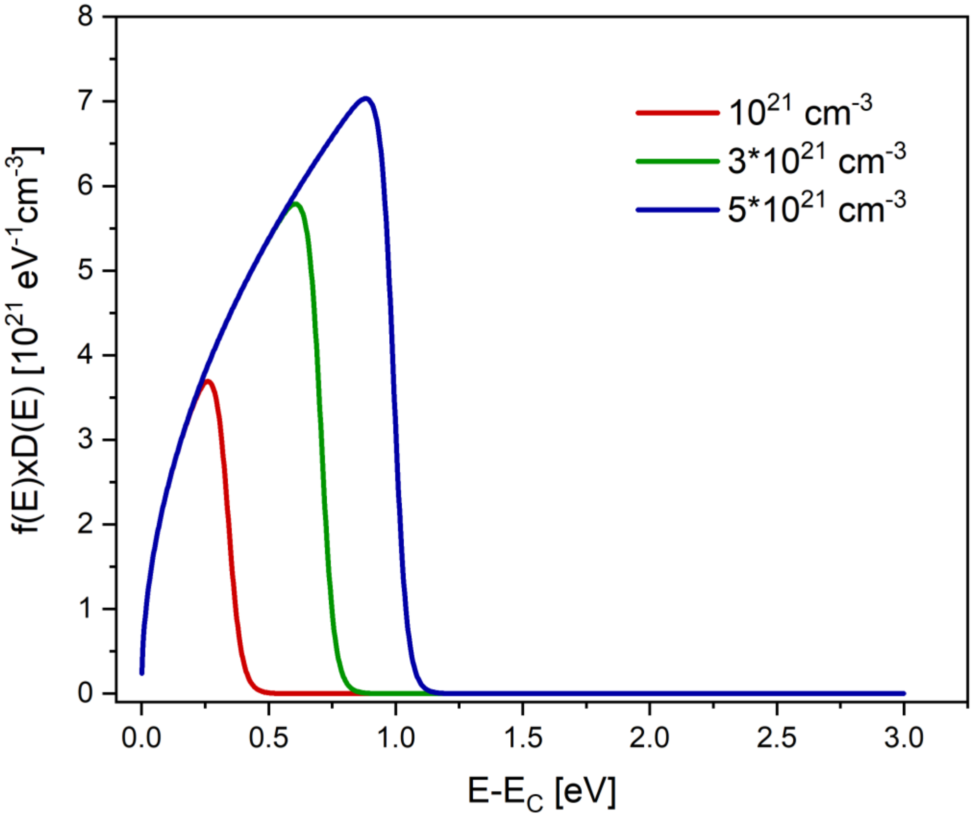

Figure SF26. Electron energy distribution at the conduction band for varying carrier densities. Above ~10^{21} cm^{-3}, the electron gas enters a highly degenerate regime, progressively filling the available density of states at specific energies and leading to the formation of an electron–hole plasma.

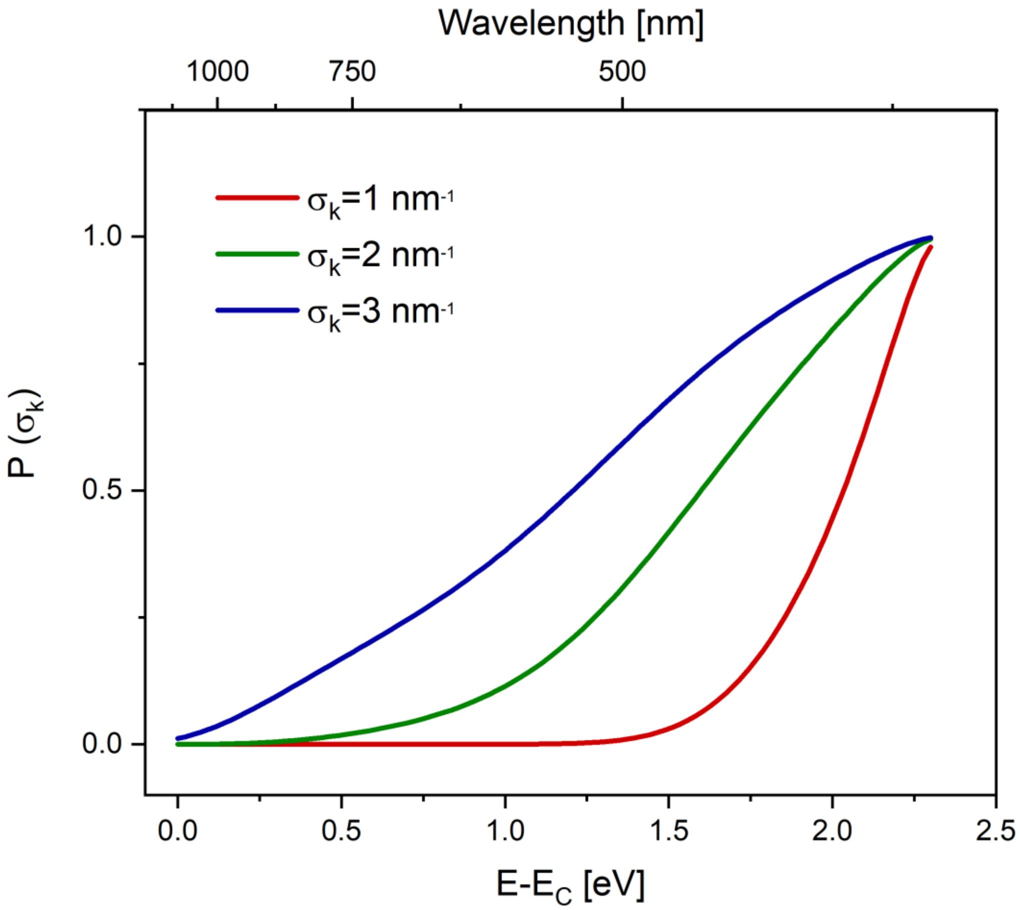

Figure SF27. The transition probability function for different photon confinement/momentum expansion levels. P(σ_k) is modeled as the projection of a Gaussian momentum distribution with width σ_k onto the parabolic dispersion of the conduction band.

We see that for an electron density of 1 x 10^{21} cm^{-3}, the conduction band is filled up to ~0.25 eV from the band edge, whereas it is filled up to ~1.0 eV when the electron density is 5 x 10^{21} cm^{-3}. In the presence of the confinement-induced radiative channel, emission can occur over the entire occupied region, which is excitation density dependent. The shape of the emission spectrum is determined by the effective overlap of two functions - conduction band occupancy (Figure SF26) and the transition probability afforded by the momentum-enhanced photonic states (Figure SF27). Importantly, the computed transition probabilities across the conduction band shown in Figure SF27 assume a Gaussian momentum distribution of σ_k variance. For 1.2 nm confiners (σ_k = 3 nm^{-1}), we expect a low transition probability near the band edge, and a higher probability closer to the excitation energy (for 532 nm this is at $E - E_C = 1.2\ eV$ in the Figure). The peak position is

largely defined by the chemical potential, i.e. effective Fermi energy for a highly degenerate electron gas, which is given as:

$$E_{F,n} - E_C = \frac{\hbar^2}{2m^*}(3\pi^2 n_{CB})^{2/3}$$

where m* is the effective mass of the electron in the conduction band and n_{CB} is the electron density. Figure SF28 shows the dependence of the effective Fermi energy on the degenerate electron density, which, in turn, depends on the laser flux. As emission from higher lying states increases with increasing excitation density, the model predicts a blue shift of the emission band. This is indeed observed, as evidenced by Figure SF29. Based on this model, we expect an emission spectrum that peaks near 650 nm for electron densities of 3 x 10^{21} eV, followed by a gradual decrease for longer wavelengths towards the band edge (1107 nm), very close what we experimentally observe.

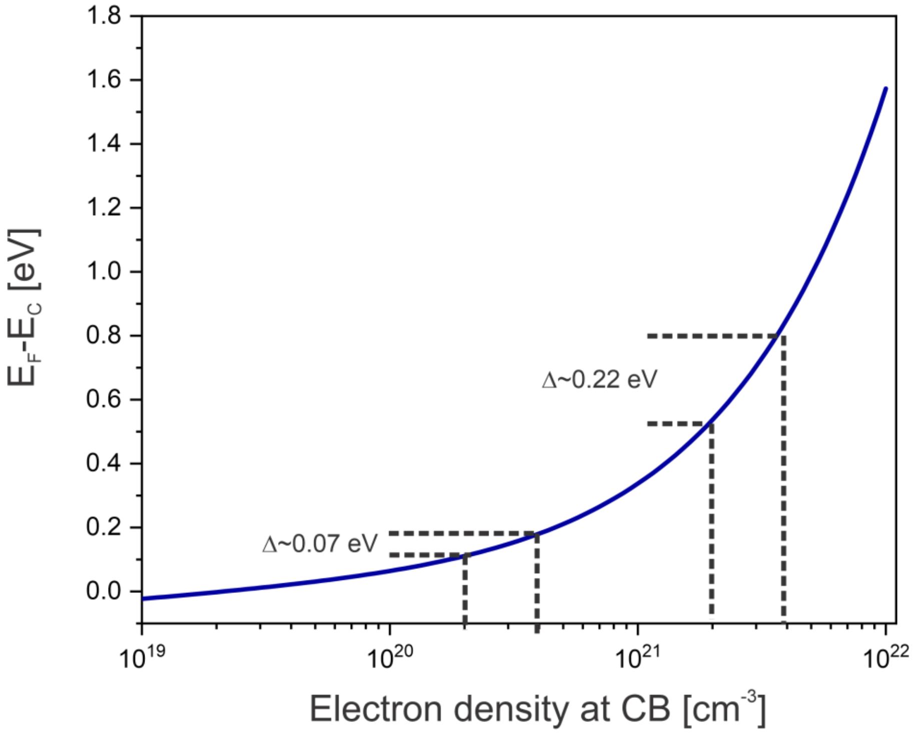

Figure SF28. Fermi energy as a function of electron gas density at the conduction band.

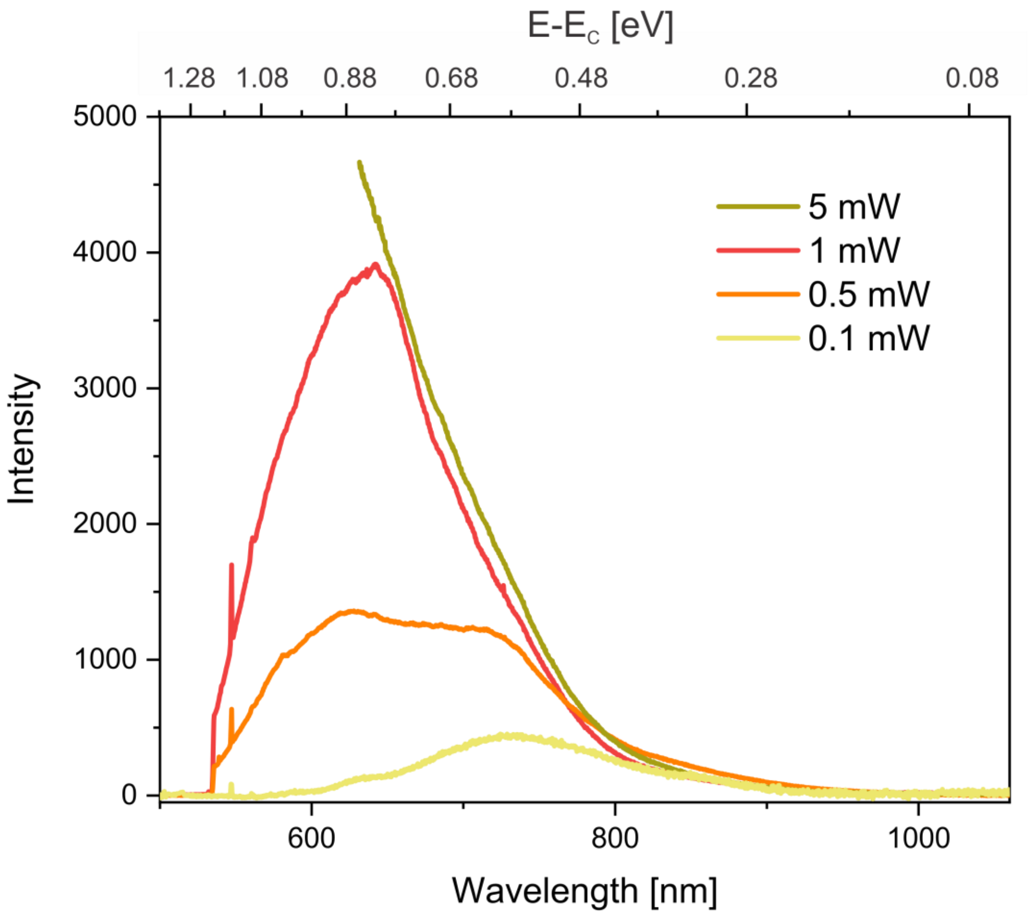

Figure SF29. Emission spectra of Si wafer decorated with 1.2 nm Au particles using different power flux. At elevated input powers (dark yellow curve), sample degradation occurs, which hinders full spectral measurements.

In the experiment, we observe a shift in the emission spectrum's center of mass in the amount of $E_{F,n} - E_C = 0.25\ eV$ when the input flux is increased by a factor of two. Comparing this situation with Figure SF29, we deduce an electron gas with a density of $>10^{21}$ cm^{-3}, which is in line with the other observations.

Part X. Particle damage under high and/or prolonged light flux.

The emission spectra of silicon decorated with 1.2 nm Au particles under 532 nm excitation are shown in Figure SF30, before and after 10 minutes of irradiation. The bright emission is significantly suppressed after extended exposure to a 1 mW light flux at the measurement point, which we attribute to the aggregation of small particles into larger clusters due to local heating. These larger clusters are too big to support optical states with sufficiently expanded momentum to facilitate new radiative transitions. This interpretation is further supported by the reappearance of the original silicon emission features, namely, the Raman signal of crystalline silicon (Figure SF30b) and phonon-assisted PL from the conduction band edge (Figure SF30c). A similar effect is observed under 785 nm excitation, as shown in Figure SF31, confirming that the suppression of new diagonal emission is a general phenomenon associated with photodamage-induced clustering.

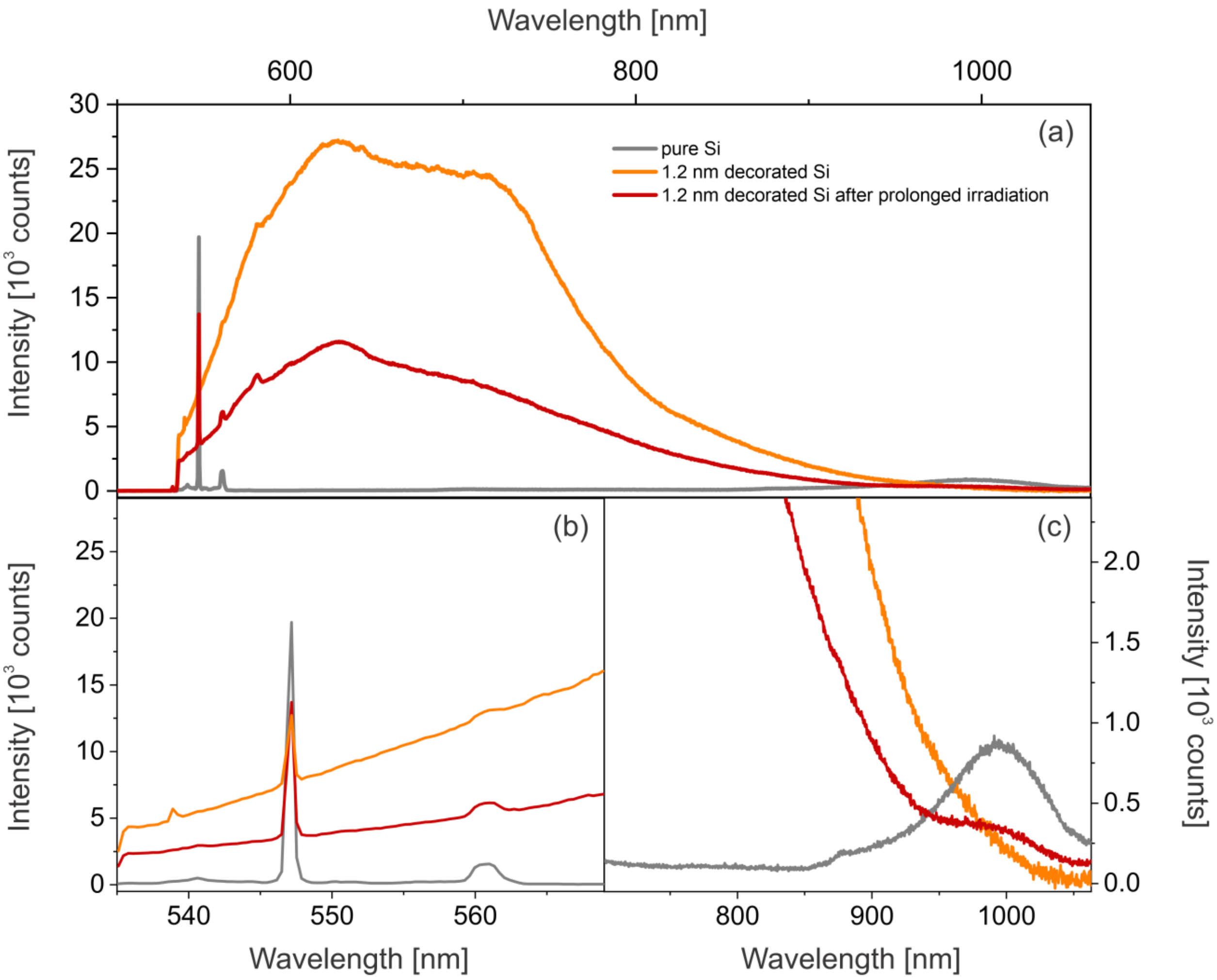

Figure SF30. (a) Emission spectrum of a silicon wafer decorated with 1.2 nm Au particles under 0.5 mW input light flux at 532 nm, shown before (orange) and after 10 minutes of prolonged irradiation under the same flux (red). (b) and (c) show the restoration of the original silicon emission features - Raman scattering (b) and phonon-assisted PL (c) from the conduction band edge - once the particles melt and aggregate into larger clusters.

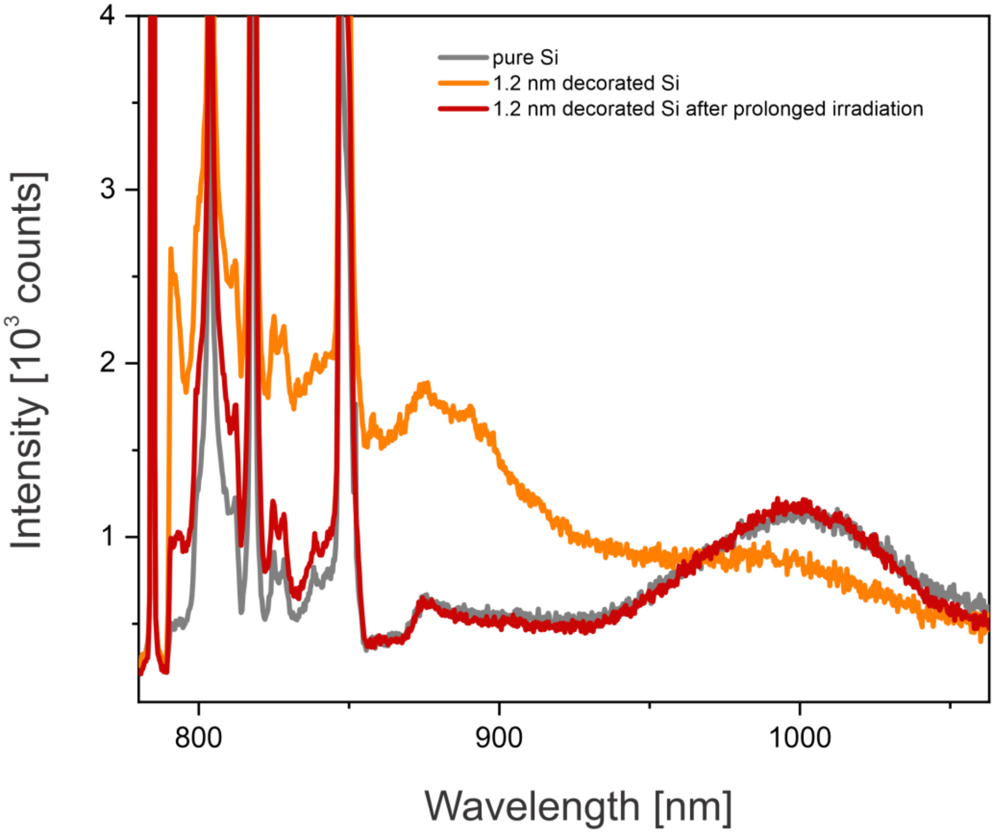

Figure SF31. (a) Emission spectrum of a silicon wafer decorated with 1.2 nm Au particles under 0.7 mW input light flux at 785 nm, shown before (orange) and after 10 minutes of prolonged irradiation under the same flux (red).

Part XI. Quantum yield estimation.

We use the photoluminescence emission from bulk, undoped GaAs as a reference signal:

$$\eta = \eta_{GaAs} \frac{I_{Si}}{I_{GaAs}} \quad ,$$

where I_{Si} and I_{GaAs} are the integrated emission intensity for Si and GaAs. This formula assumes a similar absorption value for both materials, which is reasonable since most of the excitation light is absorbed in both cases. We estimate the quantum efficiency of the new emission in silicon to be approximately $\eta \approx 0.53$.

The comparison was performed side-by-side using the same experimental setup and identical conditions, including excitation wavelength, illumination flux, collection efficiency, detection geometry, and acquisition time. The estimation does not require any correction for reflection losses, as the reflection coefficients of GaAs and Si are nearly identical in the relevant excitation range (n_{GaAs}=4.13 vs n_{Si}=4.15).

Table 1. Direct comparison with photoluminescence from bulk GaAs surface.

	**GaAs**	**Decorated Si**
Effective power used	0.5 mW	0.5 mW
Integrated emission spectrum area, *I*	764,532 counts	583,424 counts
External quantum efficiency, η	0.72, Ref. [31,32]	**~0.53**

Part XII. Visualization.

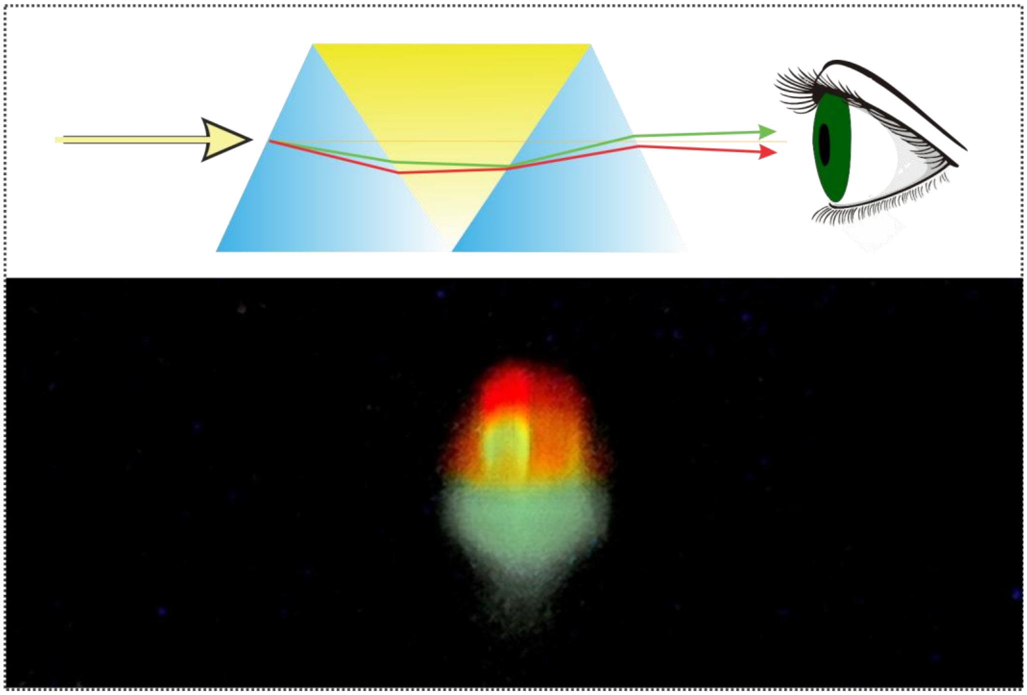

Figure SF32. The illustration shows emission viewed through an Amici prism and recorded with an iPhone camera under 532 nm excitation. 10 s integration time.

References

1 Wang, Y., Wang, M., Han, L., Zhao, Y. & Fan, A. Enhancement effect of p-iodophenol on gold nanoparticle-catalyzed chemiluminescence and its applications in detection of thiols and guanidine. *Talanta* **182**, 523-528, doi:https://doi.org/10.1016/j.talanta.2018.01.093 (2018).

2 Dong, J., Carpinone, P. L., Pyrgiotakis, G., Demokritou, P. & Moudgil, B. M. Synthesis of Precision Gold Nanoparticles Using Turkevich Method. *KONA Powder and Particle Journal* **37**, 224-232, doi:10.14356/kona.2020011 (2020).

3 Kharintsev, S. S. *et al*. Photon Momentum Enabled Light Absorption in Silicon. *ACS Nano* **18**, 26532-26540, doi:10.1021/acsnano.4c02656 (2024).

4 Noskov, A. I., Kotlyar, A. B., Potma, E. O. & Fishman, D. A. Broadband photo-and electroluminescence from silicon via momentum-expanded photonic states. *arXiv preprint arXiv:.12690* (2025).

5 Mooradian, A. Photoluminescence of Metals. *Physical Review Letters* **22**, 185-187, doi:10.1103/PhysRevLett.22.185 (1969).

6 Farrer, R. A., Butterfield, F. L., Chen, V. W. & Fourkas, J. Highly efficient multiphoton-absorption-induced luminescence from gold nanoparticles. *Nano Letters* **5**, 1139-1142 (2005).

7 Wang, H. *et al*. In vitro and in vivo two-photon luminescence imaging of single gold nanorods. *Proceedings of the National Academy of Sciences* **102**, 15752-15756 (2005).

8 Chen, W. *et al*. Intrinsic luminescence blinking from plasmonic nanojunctions. *Nature Communications* **12**, 2731, doi:10.1038/s41467-021-22679-y (2021).

9 Shalaev, V. M., Douketis, C., Haslett, T., Stuckless, T. & Moskovits, M. Two-photon electron emission from smooth and rough metal films in the threshold region. *Physical Review B* **53**, 11193-11206, doi:10.1103/PhysRevB.53.11193 (1996).

10 Imura, K., Nagahara, T. & Okamoto, H. Near-field two-photon-induced photoluminescence from single gold nanorods and imaging of plasmon modes. *The Journal of Physical Chemistry B* **109**, 13214-13220 (2005).

11 Varnavski, O., Goodson Iii, T., Mohamed, M. & El-Sayed, M. Femtosecond excitation dynamics in gold nanospheres and nanorods. *Physical Review B* **72**, 235405 (2005).

12 Tcherniak, A. *et al*. One-photon plasmon luminescence and its application to correlation spectroscopy as a probe for rotational and translational dynamics of gold nanorods. *The Journal of Physical Chemistry C* **115**, 15938-15949 (2011).

13 Fang, Y. *et al*. Plasmon emission quantum yield of single gold nanorods as a function of aspect ratio. *ACS nano* **6**, 7177-7184 (2012).

14 Yorulmaz, M., Khatua, S., Zijlstra, P., Gaiduk, A. & Orrit, M. Luminescence quantum yield of single gold nanorods. *Nano letters* **12**, 4385-4391 (2012).

15 Zhang, T. *et al*. Single bipyramid plasmonic antenna orientation determined by direct photoluminescence pattern imaging. *Advanced Optical Materials* **1**, 335-342 (2013).

16 Wu, X. *et al*. High-photoluminescence-yield gold nanocubes: for cell imaging and photothermal therapy. *ACS Nano* **4**, 113-120 (2010).

17 Park, J. *et al*. Two-photon-induced photoluminescence imaging of tumors using near-infrared excited gold nanoshells. *Opt. Express* **16**, 1590-1599 (2008).

18 Mahajan, S. *et al*. Understanding the Surface-Enhanced Raman Spectroscopy “Background”. *The Journal of Physical Chemistry C* **114**, 7242-7250, doi:10.1021/jp907197b (2010).

19 Crampton, K. T. *et al*. Ultrafast Coherent Raman Scattering at Plasmonic Nanojunctions. *The Journal of Physical Chemistry C* **120**, 20943-20953, doi:10.1021/acs.jpcc.6b02760 (2016).

20 Inagaki, M. *et al.* Electronic and vibrational surface-enhanced Raman scattering: from atomically defined Au(111) and (100) to roughened Au. *Chemical Science* **11**, 9807-9817, doi:10.1039/D0SC02976A (2020).
21 Kamimura, R., Kondo, T., Motobayashi, K. & Ikeda, K. Surface-Enhanced Electronic Raman Scattering at Various Metal Surfaces. **259**, 2100589, doi:https://doi.org/10.1002/pssb.202100589 (2022).
22 Beversluis, M. R., Bouhelier, A. & Novotny, L. J. P. R. B. Continuum generation from single gold nanostructures through near-field mediated intraband transitions. **68**, 115433 (2003).
23 Loirette-Pelous, A. & Greffet, J.-J. Theory of Photoluminescence by Metallic Structures. *ACS Nano* **18**, 31823-31833, doi:10.1021/acsnano.4c07637 (2024).
24 Rangel, T. *et al.* Band structure of gold from many-body perturbation theory. *Physical Review B* **86**, 125125, doi:10.1103/PhysRevB.86.125125 (2012).
25 Ndione, P. D., Gericke, D. O. & Rethfeld, B. Optical Properties of Gold After Intense Short-Pulse Excitations. **10**, doi:10.3389/fphy.2022.856817 (2022).
26 Zhang, P., Feist, J., Rubio, A., García-González, P. & García-Vidal, F. J. Ab initio nanoplasmonics: The impact of atomic structure. *Physical Review B* **90**, 161407, doi:10.1103/PhysRevB.90.161407 (2014).
27 Urbieta, M. *et al.* Atomic-Scale Lightning Rod Effect in Plasmonic Picocavities: A Classical View to a Quantum Effect. *ACS Nano* **12**, 585-595, doi:10.1021/acsnano.7b07401 (2018).
28 Baumberg, J. J. Picocavities: a Primer. *Nano Letters* **22**, 5859-5865, doi:10.1021/acs.nanolett.2c01695 (2022).
29 Lee, J., Crampton, K. T., Tallarida, N. & Apkarian, V. A. Visualizing vibrational normal modes of a single molecule with atomically confined light. *Nature* **568**, 78-82, doi:10.1038/s41586-019-1059-9 (2019).
30 Zhang, Y. *et al.* Visually constructing the chemical structure of a single molecule by scanning Raman picoscopy. *National Science Review* **6**, 1169-1175, doi:10.1093/nsr/nwz180 %J National Science Review (2019).
31 Johnson, S. R., Ding, D., Wang, J.-B., Yu, S.-Q. & Zhang, Y.-H. Excitation dependent photoluminescence measurements of the nonradiative lifetime and quantum efficiency in GaAs. *Journal of Vacuum Science & Technology B: Microelectronics and Nanometer Structures Processing, Measurement, and Phenomena* **25**, 1077-1082, doi:10.1116/1.2720864 %J Journal of Vacuum Science & Technology B: Microelectronics and Nanometer Structures Processing, Measurement, and Phenomena (2007).
32 Wang, C., Li, C.-Y., Hasselbeck, M. P., Imangholi, B. & Sheik-Bahae, M. Precision, all-optical measurement of external quantum efficiency in semiconductors. *Journal of Applied Physics* **109**, doi:10.1063/1.3580259 (2011).
